# Genome‐scale phylogeography resolves the native population structure of the Asian longhorned beetle, *Anoplophora glabripennis* (Motschulsky)

**DOI:** 10.1111/eva.13381

**Published:** 2022-06-07

**Authors:** Mingming Cui, Yunke Wu, Marion Javal, Isabelle Giguère, Géraldine Roux, Jose A. Andres, Melody Keena, Juan Shi, Baode Wang, Evan Braswell, Scott E. Pfister, Richard Hamelin, Amanda Roe, Ilga Porth

**Affiliations:** ^1^ Institut de Biologie Intégrative et des Systèmes Université Laval Québec Québec Canada; ^2^ Département des sciences du bois et de la forêt Université Laval Québec Québec Canada; ^3^ Forest Pest Methods Laboratory Plant Protection and Quarantine Science and Technology Animal and Plant Health Inspection Service United States Department of Agriculture Buzzards Bay Massachusetts USA; ^4^ Centre d'Écologie Fonctionnelle et Évolutive Université Montpellier Montpellier France; ^5^ Institut National de la Recherche Agronomique UR633 Zoologie Forestière Orléans France; ^6^ 27057 COST Université d’Orléans Orléans France; ^7^ Department of Ecology and Evolutionary Biology Cornell University Ithaca New York USA; ^8^ United States Department of Agriculture Forest Service Northern Research Station Hamden Connecticut USA; ^9^ 12380 Key Laboratory for Silviculture and Conservation of Ministry of Education Beijing Forestry University Beijing China; ^10^ Insect Management and Molecular Diagnostics Laboratory Plant Protection and Quarantine Science and Technology, Animal and Plant Health Inspection Service United States Department of Agriculture Edinburg Texas USA; ^11^ 4440 Department of Forest and Conservation Sciences The University of British Columbia Vancouver British Columbia Canada; ^12^ Canadian Forest Service Great Lakes Forestry Centre Natural Resources Canada Sault Ste. Marie Ontario Canada

**Keywords:** gene flow, genotyping‐by‐sequencing, glycerol, insect pest, invasion history, population assignment

## Abstract

Human‐assisted movement has allowed the Asian longhorned beetle (ALB, *Anoplophora glabripennis* (Motschulsky)) to spread beyond its native range and become a globally regulated invasive pest. Within its native range of China and the Korean peninsula, human‐mediated dispersal has also caused cryptic translocation of insects, resulting in population structure complexity. Previous studies used genetic methods to detangle this complexity but were unable to clearly delimit native populations which is needed to develop downstream biosurveillance tools. We used genome‐wide markers to define historical population structure in native ALB populations and contemporary movement between regions. We used genotyping‐by‐sequencing to generate 6102 single‐nucleotide polymorphisms (SNPs) and amplicon sequencing to genotype 53 microsatellites. In total, we genotyped 712 individuals from ALB’s native distribution. We observed six distinct population clusters among native ALB populations, with a clear delineation between northern and southern groups. Most of the individuals from South Korea were distinct from populations in China. Our results also indicate historical divergence among populations and suggest limited large‐scale admixture, but we did identify a restricted number of cases of contemporary movement between regions. We identified SNPs under selection and describe a clinal allele frequency pattern in a missense variant associated with glycerol kinase, an important enzyme in the utilization of an insect cryoprotectant. We further demonstrate that small numbers of SNPs can assign individuals to geographic regions with high probability, paving the way for novel ALB biosurveillance tools.

## 1 INTRODUCTION

Globalization has increased the spread and establishment of invasive species throughout the world, causing irreversible damage to forest ecosystems (Millar & Stephenson, 2015; Seebens et al., 2017). As it is difficult and expensive to manage established invasive species, the prevention and early detection of new invasive species and populations are the cornerstones of an effective management response (Reaser et al., 2020). In this respect, biosurveillance represents a knowledge framework that facilitates early detection and rapid response to new invasive threats, reducing the risk and impact of invasive species in novel habitats (Blackburn et al., 2020).

Tracking introduction pathways and identifying the geographic sources of alien pests is an essential component of an invasive species biosurveillance pipeline (Bilodeau et al., 2019; Cristescu, 2015). Knowledge of invasion sources allows management responses to focus on high‐risk points of entry and routes of spread, which facilitates invasive species monitoring, trade negotiations, and future risk assessments (Bilodeau et al., 2019). However, this approach requires clear delimitation of an invasive species’ population structure in its native range so that intercepted individuals can be genetically assigned to a source population (Hamelin & Roe, 2020; Manel et al., 2005; Roe et al., 2019). Thus, accurately characterizing the population structure and genetic diversity within the native range of an invasive species is an essential step toward effective biosurveillance and management of high‐risk pests.

**FIGURE 1:**
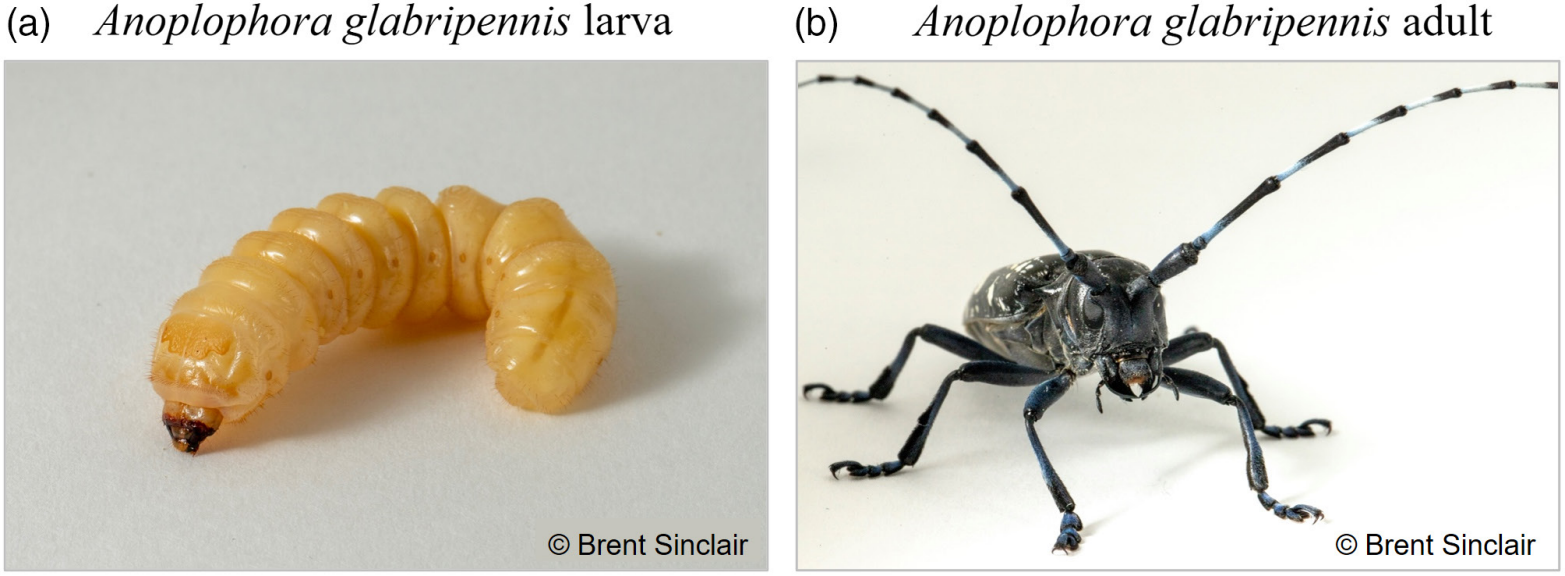
Larva and adult of *Anoplophora glabripennis*, the Asian longhorned beetle. Pictures were kindly provided by Dr. Brent Sinclair

The Asian longhorned beetle (ALB, Figure 1) (Coleoptera: Cerambycidae: *Anoplophora glabripennis* (Motschulsky)) is a wood‐boring insect native to China and the Korean peninsula that has become a highly destructive invasive pest and poses a global threat to temperate broadleaved forests (Lingafelter & Hoebeke, 2002). This species is broadly distributed in East Asia, spanning much of the temperate forested regions in mainland China and the Korean Peninsula and extending south of 35°N into subtropical regions in China and it broadly overlaps with the citrus longhorned beetle (CLB, *A*. *chinensis* (Forster)), another important invasive pest. Within its native range, ALB is considered an important forest pest, damaging and killing many tree species, such as poplar (*Populus* spp.), willow (*Salix* spp.), and maple (*Acer* spp.) (Lingafelter & Hoebeke, 2002; Sjöman et al., 2014). In less than two decades, the area that ALB populations occupied had increased by seven‐fold, and by 1994, had impacted over 333,000 ha of broadleaf forests within China (Luo et al., 2000).

ALB was initially detected in North America in 1996 and then in 2001 in Europe where it has established and spread, causing severe ecological and economic losses in these newly invaded regions (Meng et al., 2015). ALB larvae tunnel and feed within the host xylem, making visual detection challenging. Wood damaged by ALB is inferior in quality and is often used to construct solid wood packaging material (e.g., pallets, crates, dunnage) which is then used to transport goods. Indeed, infested wood packaging material is considered a crucial pathway for ALB’s invasion, facilitated by increasing globalization and trade (Wu et al., 2017). In response to the ALB invasion, the Food and Agriculture Organization (FAO) established International Standards for Phytosanitary Measures (ISPM) 15, which set minimum heat and fumigation treatments for wood packaging material used in global trade to render potential invasives nonviable (FAO, 2002). However, prior to global acceptance of ISPM 15, ALB was transported throughout the world and introduced to many new broadleaf ecosystems. Despite these new phytosanitary protocols, ALB continues to pose a significant threat to temperate forests outside its native range with recent interceptions and introductions (Haack, 2020; Pedlar et al., 2020) throughout the invaded range. Therefore, biosurveillance tools are needed to manage the threat posed by ALB and identify the regional source(s) of invasion.

Complex biogeographic patterns are typical among native species within China (Lyu et al., 2020). While East Asia is known to maintain complex, deeply divergent population structure (Qiu et al., 2011), this historic structuring can be eroded with contemporary migration between distinct populations, often facilitated through anthropogenic movement of individuals beyond their ability to disperse naturally (Eloff et al., 2020). In ALB’s native range, population differentiation is driven by both evolutionary and anthropogenic processes (Colunga‐Garcia et al., 2010; Huang et al., 2020; Shatz et al., 2013). Indeed, forestry practices in northern China from the 1950s to1970s, including the Three‐North Shelter reforestation project which is the largest artificial forest worldwide, created forest conditions that caused ALB populations to irrupt and spread beyond their historic range. Previous studies not only reported moderate levels of population differentiation but also found high levels of admixture (Carter et al., 2009; Javal et al., 2019, 2019,2019, 2019). These authors hypothesized that this complex population structure was due to widespread contemporary movement of ALB associated with movement of infested wood out of China's Three‐North Shelter reforestation plantation. This assisted migration caused gene flow between different populations, blurring the historic population structure. However, these previous methods used random amplified polymorphic DNA markers (An et al., 2004), mitochondrial DNA (Carter et al., 2009, 2010; Javal et al., 2019b), and small sets of microsatellite markers (Carter et al., 2010; Javal et al., 2019a), which lack the resolution needed to describe complex patterns of historic population structure and contemporary gene flow and, therefore, more information‐rich marker systems are needed.

Genomic data provide unparalleled insights into the invasion history of an invasive pest (Hamelin & Roe, 2020). These data can quantify variation among populations and allow us to identify individuals that share common ancestors and geographic origins. Genomic technologies, such as reduced representation libraries (Altshuler et al., 2000; Van Tassell et al., 2008), provide access to variable genetic loci throughout the genome. Genotyping‐by‐Sequencing (GBS) (Elshire et al., 2011), double digest restriction‐site associated DNA sequencing (ddRADseq) (Peterson et al., 2012), and DNA simple sequence repeats (microsatellites)‐based genotyping (Jarne & Lagoda, 1996; Luikart et al., 2003) have been used to characterize population structure in a range of invasive insect pests, such as *Lymantria dispar* from Asia (Picq et al., 2018; Wu et al., 2020), the Asian granulated ambrosia beetle *Xylosandrus crassiusculus* (Storer et al., 2017), and the fall webworm *Hyphantria cunea* (Cao et al., 2016). With these approaches, high numbers of polymorphic markers can resolve fine‐scale patterns of genomic differentiation and assign invasive samples to distinct populations with high levels of accuracy. Furthermore, genomic markers were able to identify signatures of population expansion within the invaded range, providing further insight into the patterns of invasion and spread within invasive pest populations (Cao et al., 2016; Picq et al., 2018; Storer et al., 2017; Wu et al., 2020).

In this study, we comprehensively defined the native ALB population structure and quantified the scale of contemporary gene flow in this species by leveraging the informative power of genome‐wide molecular markers. To complement and cross‐validate our inferred population structure, we used two sets of independent data: SNPs obtained from GBS, and microsatellites obtained from amplicon sequencing. First, we generated SNPs using a reduced representation library approach to characterize genomic diversity and population structure in ALB’s native range. We then validated our proposed population structure with an independent set of insect samples and 53 microsatellites developed with ddRADseq and genotyped through amplicon sequencing. Using our panel of informative SNPs, we delineate ALB population structure, reconstruct population history scenarios to describe the observed population structure, and identified loci that were under selection that may show adaptive significance within ALB populations. We identified loci that proved diagnostic for our delineated ALB populations and provided high assignment accuracy for downstream use in an amplicon‐based ALB biosurveillance tool for this high‐risk invasive forest pest.

## 2 MATERIALS AND METHODS

### 2.1 ALB sampling and DNA preparations for SNP genotyping

**FIGURE 2:**
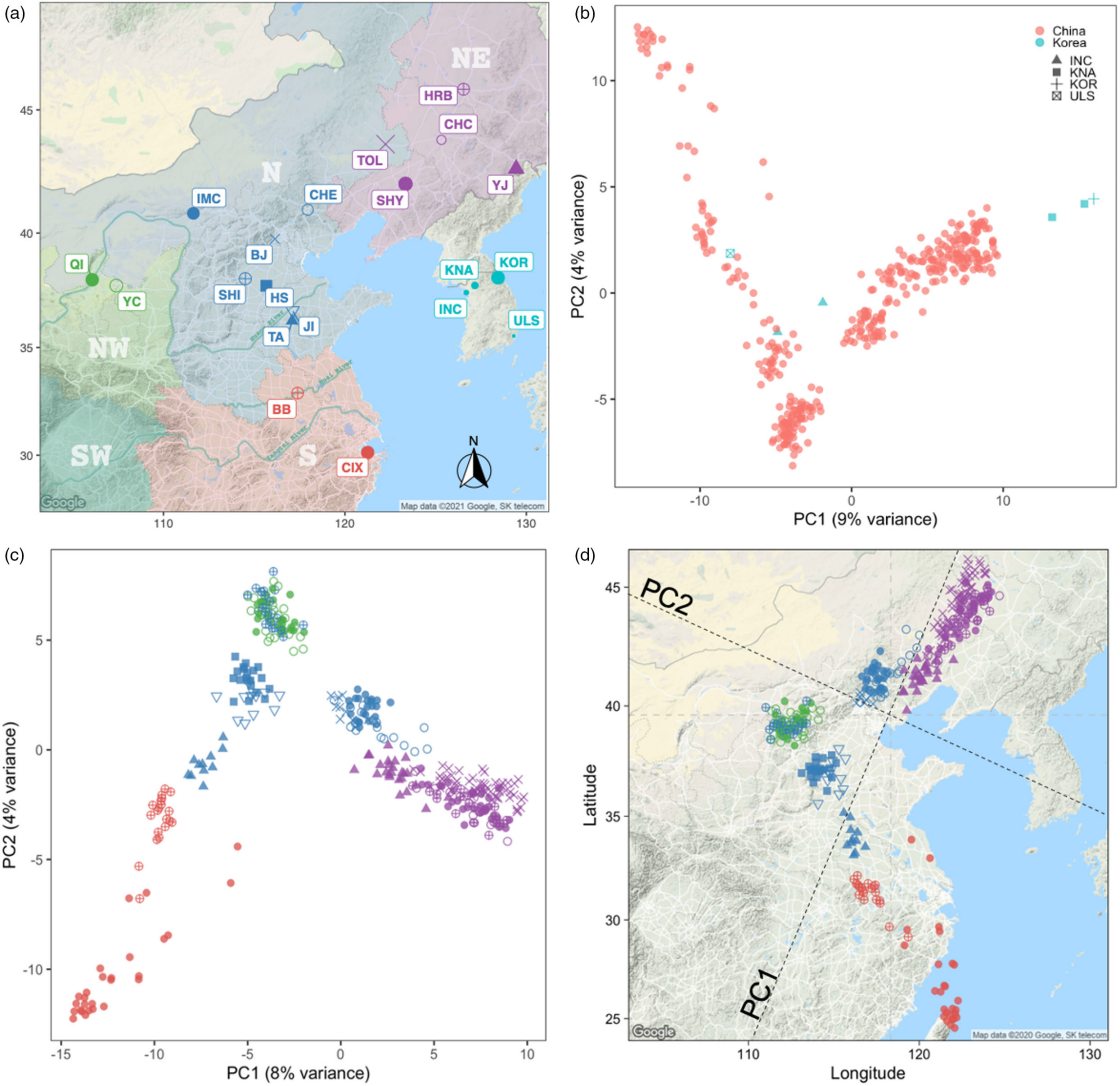
Asian longhorned beetle sampling and Principal Component Analysis (PCA). (a) Sampling map of *Anoplophora glabripennis* (ALB). The codes for the populations are shown in Table 1. The icon size of each population is relative to the sample size. We divided the ALB sampling locations in China into four areas, according to biogeography, distinguished by different colors: N, north China (blue); NW, northwest China (green); NE, northeast China (lilac); S, south China (red); in addition to China, Korea (turquois), was also sampled for ALB. Each point with a different color or shape represents a distinct sampling location. TOL is treated as a NE population given its geographical location. The three rivers shown are Huang River, Huai River, and Yangtze River from north to south. (b) PCA of ALB populations in the native range based on 6102 SNPs. (c) PCA of Chinese ALB populations. (d) Procrustes‐transformed PCA of Chinese ALB populations based on the geographical coordinates of each sample. The colors and symbols correspond to those shown in (a)

We collected 480 samples from 20 sites across China and South Korea (Figure 2a; sampling details in Table S1), which covered c. 40% of ALB’s distribution in the native range according to its most current distribution record (CABI, 2021; Yan, 1985). Our samples also covered several Three‐North regions, including Heilongjiang, Jilin, Liaoning, Hebei, Beijing City, Inner Mongolia, and Ningxia. These represent the major biogeographic regions in northern China, with the Greater Khingan Range dividing the Northeast and the Helan Mountains dividing the Northwest (He et al., 2017; Huang et al., 2012). In the south, the Huai River basin is a major transition zone between northern and southern climatic regions as it approximates the 0°C January isotherm (Shi et al., 2014). The Yangtze River is also recognized as an important North‐South boundary within the region. Based on the regions separated by these major geographic barriers, we defined the three regions located north of the Huai River as “Northeast (NE),” “North (N),” and “Northwest (NW)”, respectively, and the one southern region, where ALB sampling occurred, “South (S)”. Bengbu (BB) is located within the Huai River basin and was therefore included in the “South” region together with Cixi (CIX).

Before DNA isolation, 95% ethanol was used to remove any contaminations from the beetle's surface. For each sample, a single leg or larval tissue was cut into smaller pieces, and the tissue was ground using a mixer mill (Retsch MM400, Germany) at 29 Hz for 1 min. DNA was extracted using the DNeasy 96 Blood &Tissue Kit (Qiagen) following the manufacturer's instructions. The quality and quantity of DNA samples were assessed using the NanoDrop ND‐1000 spectrophotometer and Qubit 2.0 Fluorometer, respectively. For each sample, a total of 1μg or 2μg DNA was prepared for sequencing.

### 2.2 Genome‐wide SNP markers: genotyping and analysis

GBS libraries were prepared and sequenced at the Institut de Biologie Intégrative et des Systèmes (IBIS; Université Laval, Quebec City, Canada) with the Ion Proton sequencer (Thermo Fisher Scientific, Waltham, MA, USA) following the procedure described in detail by Abed et al. (2019). We modified their protocol by adding NsiI to the PstI/MspI double digest to create a triple digest library and adopted double‐size selection steps to increase the efficiency and accuracy of marker discovery (Bayona‐Vásquez et al., 2019; Peterson et al., 2012).

The Fast‐GBS pipeline v1.0 was adopted to process raw sequencing reads (Torkamaneh et al., 2017). Within this pipeline, SABRE 1.0 was used to demultiplex barcoded reads into separate files (Joshi, 2011), Cutadapt 2.1 to remove adapter sequences (Martin, 2011), Burrows‐Wheeler Aligner (BWA) 0.7.17 (Li & Durbin, 2010) to align reads to the ALB reference genome which consists of 10,474 scaffolds (GCA_000390285.1) (McKenna et al., 2016), Samtools 1.8 to convert sam files to bam format and index (Li et al., 2009), and lastly, PLATYPUS 0.8.1.1 to call the SNP variants (Rimmer et al., 2014). The resulting SNP variants were filtered using VCFtools 0.1.16 (Danecek et al., 2011). Basic filters were applied to retain only biallelic SNPs, remove indels and variants with a FILTER flag other than PASS, and remove loci with more than 50% missing data. Samples with >60% missing data were removed. Finally, only loci with read depth >5, minor allele frequency (MAF) >0.05, minor allele count (MAC) >3, and missing data per site <10% were retained. We estimated relatedness between samples through the PLINK method of moment (MoM) using the "snpgdsIBDMoM" function in SNPRelate (Zheng et al., 2012) implemented in R (Team, 2015). Samples with an identity‐by‐descent coefficient exceeding 0.25, which are considered full sibs, were removed. However, genetically related individuals from Korea, where we had limiting sampling, were initially retained for analyses to explore our Korean samples’ overall genetic diversity and compare them to the Chinese populations. Following these comparisons, these analyses were repeated without the related Korean individuals. The SNP variant filtering pipeline is outlined in Table S2.

#### 2.2.1 Genetic diversity

We calculated population genetic indices such as nucleotide diversity (π), observed heterozygosity (*H_o_*), and expected heterozygosity (*H_e_*) using the function “populations” in STACKS v2.3e (Catchen et al., 2013). We calculated the observed heterozygosity for each individual via VCFtools‐0.1.16 and the pairwise *F*_ST_ between populations via GenoDive 3.04 (Meirmans, 2020). The relationships of *H_e_* or *H_o_* with latitude or longitude were assessed using stat_cor() in R (Team, 2015).

#### 2.2.2 Population structure

We assessed population structure among native ALB populations using three complementary multivariate ordination methods: principal component analysis (PCA) (Price et al., 2006), discriminant analysis of principal components (DAPC) (Jombart et al., 2010), and spatial principal component analysis (sPCA) (Jombart et al., 2008). We used PCA to demonstrate genetic similarities among individuals and then DAPC to delimit distinct genetic clusters. Finally, we used sPCA to explicitly incorporate spatial information to quantify geographic patterns of genetic variation. We performed all three analyses using adegenet v2.1.2 (Jombart, 2008). For PCA analysis, we used the "glPca" function with retaining 20 PCs. For DAPC analysis, we first ran the "find.clusters" function to determine the number of groups that best summarize variations in the data running from *K* = 1 to 16. We then used the "dapc" function while retaining 250 PCs and five discriminant analysis eigenvalues. In the sPCA analysis, we ran the "spca" function by choosing a Delaunay triangulation as a connection network and kept the first positive eigenvalue. To assess similarities between geographical and genetic distributions, we performed a Procrustes analysis and a Mantel test (Lisboa et al., 2014). We used the “procrustes” function in the R package MCMCpack 1.4–7 (Martin et al., 2011) to perform a Procrustes transformation analysis on the first two PCs identified in the PCA analysis, in which the PCA coordinates were scaled to geographical coordinates. We determined the Pearson correlation coefficient between the pairwise genetic distance (*F*_ST_) and the geographical distance between populations using stat_cor() in R.

Based on the distinct groups identified by DAPC analysis, we performed an analysis of molecular variance (AMOVA) using an infinite allele model in GenoDive 3.04 (Meirmans, 2020) to separate total genetic variance into among‐individuals within‐populations, among‐populations within‐groups, and among‐groups covariance components.

Contemporary movement of ALB was previously hypothesized by authors (Carter et al., 2009; Javal, Lombaert, et al., 2019), therefore, we wanted to quantify levels of admixture within each population. We used a sparse non‐negative matrix factorization (sNMP) approach to estimate individual ancestry coefficients using LEA 2.0 (Frichot & François, 2015). This method allowed us to estimate homozygote and heterozygote frequencies and avoid the assumption of Hardy–Weinberg equilibrium (HWE) (Frichot et al., 2014). We ran ten replicates with K range 1 to 16 using the “snmf” function. It used a cross‐validation approach to estimate the entropy of each *K*, where the minimum *K* value is the best estimate. We then ran “snmf” again with the ascertained *K* and an alpha‐value (regularization parameter) of 100 to estimate the individual ancestry coefficients. We also computed a maximum likelihood (ML) phylogeny using 1000 bootstrap replicates under the GTRGAMMA model in RAxML v8.2.9 (Stamatakis, 2014). We visualized the ML tree with FigTree v1.4.4 (http://tree.bio.ed.ac.uk/software/figtree/).

#### 2.2.3 Gene flow and population history models

We calculated recent gene flow among populations with a Bayesian Markov Chain Monte Carlo (MCMC) resampling method in BayesAss 3.04, which estimates recent migration rates between populations as the proportion of migrants per generation (Mussmann et al., 2019). First, we ran 1,000,000 iterations without burn‐in and then adjusted the parameter for allele frequencies to 0.3 to align with the suggested acceptance rate of 20%~60% (Wilson & Rannala, 2003). Then, we performed three independent runs using 10 million iterations with a burn‐in of one million and a sampling interval of 1000. We used the default settings (0.1) for the mixing parameters associated with proposed moves (i.e., inbreeding coefficient and migration rates). We assessed convergence of the estimated parameters by plotting the combined trace plot and the probability distribution using Tracer 1.7.1 (Rambaut et al., 2018).

We used Bayesian inference with the structured coalescent estimator Migrate 4.4.4 (Beerli et al., 2019) to test population history models for the four regions N, S, NE, and NW based on our population structure results. Thus, the following different population scenarios were evaluated: seven scenarios (Figure S1, models 1–7) that assumed the most recent common ancestry occurred in the N or NW regions based on their high genetic diversity, and among which, four of the scenarios (Figure S1, models 2–5) assumed that the S and NE populations were from different sources; we included two admixture models (Figure S1, models 8–9) to assess whether population admixture has occurred within the North region, given our admixture result. We randomly selected 20 individuals from each region and ran the models with a strictly filtered SNP dataset that contained no missing data (*n* = 422 SNPs). We used one long chain with four replicates for each run with 1,000,000 iterations and a burn‐in of 10,000. We compared the models using the log marginal likelihood (Beerli et al., 2019). To further estimate effective population sizes, divergence time, and migration rates of the optimal model identified with Migrate, we used an alternative coalescent‐based simulation method, Fastsimcoal 2.6 (Excoffier & Foll, 2011). We applied easySFS.py (https://github.com/isaacovercast/easySFS) to generate the joint site frequency spectrum (SFS). We performed 100 independent parameter estimations to obtain the maximum composite likelihood of the joint SFS, each run with 100,000 iterations and 40 cycles of expectation conditional maximization (ECM) (Lanier et al., 2015) using a maximum likelihood method. The mutation rate μ was set to 2.9e^−09^ per base and per generation based on *Heliconius melpomene* (Keightley et al., 2015), assuming a 1‐year life cycle (generation time) for ALB (Hu et al., 2009). The best run was selected based on the highest maximum likelihood. We computed the 95% confidence intervals of the parameter estimates of the best run by simulating 50 SFS of the estimates and re‐estimating parameters each time (Lanier et al., 2015; Zhao et al., 2019b). PGDSpider 2.1.1.5 was used to convert the vcf file to the required file formats for the above analyses (Lischer & Excoffier, 2012).

#### 2.2.4 Selection detection

We used the *F*_ST_‐based genome scan method OutFLANK v0.2 (Whitlock & Lotterhos, 2015) to detect loci putatively under selection. It calculates the likelihood based on a trimmed distribution of *F*_ST_ values to infer the *F*_ST_ distribution for neutral markers. We used a *q*‐value of 0.05 to identify outlier loci. We also used *fsthet* v1.0.1 (Flanagan & Jones, 2017) as an alternative method to detect loci under selection in R. It calculates *F*_ST_ and expected heterozygosity values for individual SNPs, from which it determines smoothed quantiles to identify loci with elevated or low‐lying *F*_ST_ values relative to their heterozygosity. Here, we designated a confidence level of 95% to detect *F*_ST_ outliers. We annotated outlier SNPs using SnpEff v5.030 (Cingolani et al., 2012). The SnpEff database for ALB was built manually with the reference genome GCA_000390285.1 (McKenna et al., 2016). The flanking sequence for each locus identified was compared to the NCBI database using blastn to identify similar insect genes (Altschul et al., 1990), and details for the annotated regions were derived from insect‐based records from the Gene Ontology database (Ashburner et al., 2000; Gene Ontology Consortium, 2021), UniProt database (UniProt Consortium, 2021), and FlyBase (Larkin et al., 2021) to determine molecular function and biological processes. We identified a potentially adaptive outlier SNP within glycerol kinase and conducted a phylogenetic analysis for related insect genes using the Neighbor‐joining method with 1000 bootstraps in the MEGA X software (Kumar et al., 2018). The allele frequencies of the candidate SNP across all populations were visualized in R (Team, 2015).

### 2.3 Microsatellite discovery and analysis

To independently assess population structure with a different set of data, we genotyped 232 new ALB specimens collected independently from 16 locations in China (Table S3) within the biogeographic regions (i.e., NW, N, NE, S) using highly polymorphic microsatellite markers. Here, we sampled within the same regions, but with limited overlap with localities of the GBS study, with only five duplicated sampling locations, Beijing (BJ), Hengshui (HS), Cixi (CIX), Tongliao (TOL), and Yanchi (YC). DNA was extracted using the same protocol outlined above. Following DNA extraction, we mined polymorphic microsatellite motifs from clean reads obtained from ddRAD sequencing, based on a library of 25 freshly preserved specimens. For ddRAD sequencing, we used restriction enzymes *SbfI* and *MspI* while simultaneously ligating P1 (*SbfI*) and P2 (*MspI*) adaptors to the fragmented DNA and targeting large fragments (sizes selected for 350–550 bp) for 2 × 300 bp paired‐end sequencing on an Illumina Miseq platform (Cornell University's Biotechnology Resource Center). After an initial quality check using FastQC, raw Illumina reads were assembled into contigs (unique consensus sequences from multiple reads) using NGen (v.11. DNASTAR. Madison, WI) and default parameters. The resulting contigs were used as the reference file to re‐align all raw Illumina reads to assess coverage and the presence of microsatellite loci for each contig.

We assembled over 26,500 contigs and discovered around 1300 nonduplicated microsatellite loci, including dimers, trimers, and tetramers. Only tetramers were developed for the subsequent multiplex assay because they normally are less prone to stuttering caused by Taq polymerase slippage (DeWoody et al., 2006). Primers were designed for 69 tetramer microsatellite loci for genotyping using BatchPrimer3 (You et al., 2008) with default parameters. They were divided into four multiplexes. PCR conditions consisted of 1 min initial denaturation at 94°C, followed by 40 cycles with 15 s denaturing at 94°C, 15 s annealing at 55°C, 30 s extension at 72°C, and a final extension time of 10 min at 72°C. Raw genotypic data were trimmed to exclude microsatellite loci with >20% missing data and specimens with >8 (15%) missing loci. The final dataset included 53 microsatellite loci (primer sequences available in Table S4) for 202 individuals (1–32 individuals per location, average 12.6). We assessed linkage disequilibrium between all pairs of microsatellite loci in the R package *genepop* 1.1.7 (Rousset, 2008) to avoid genetically linked loci.

Mean number of alleles per locus (*A_n_*), average gene diversity over loci (D), observed heterozygosity (*H_o_*), and expected heterozygosity (*H_e_*) under HWE were estimated in Arlequin 3.5 (Excoffier & Lischer, 2010). The probability test for deviation from HWE was calculated in the R package *genepop* 1.1.7 (Rousset, 2008). We used the R package *PopGenReport* 3.0.4 (Adamack & Gruber, 2014) to estimate mean allelic richness (*A_s_*) for each location and pairwise *F*_ST_ after correcting for sample size differences. Samples with fewer than five specimens were omitted from the estimation.

We applied DAPC to characterize genetic clusters using Ward's hierarchical clustering method (Ward, 1963). We further assessed the distribution of genetic variance between and within genetic clusters as assessed through an AMOVA based on the clusters according to our DAPC results. Statistical significance of covariance associated with each hierarchical level was calculated with 1000 permutations.

### 2.4 Congruence between genome‐wide marker sets

To assess the congruence between the two marker sets, we performed a Mantel test using a Monte‐Carlo method on the pairwise F_ST_ generated from SNPs and microsatellites in ade4 (Chessel et al., 2004). We used F_ST_ values calculated from the five sampling locations (i.e., BJ, HS, CIX, TOL, and YC) present in both datasets. We performed the ‘mantel.rtest’ analysis on the two *F*_ST_ matrices with 9999 permutations.

### 2.5 Population assignment

To identify markers for downstream diagnostic tool development, we wished to identify SNPs that could accurately assign individuals to their source populations. To do so, we used Mycorrhiza 0.0.28, a genotype assignment software that employs machine learning and phylogenetic networks to identify informative SNPs that can assign individuals to their source population (Georges‐Filteau et al., 2020). The SNPs were ranked by discriminatory power based on mutual information. Mycorrhiza works in two steps. First, it generates a pairwise genetic distance matrix from the genotype data and uses it to construct a phylogenetic split system using the Neighbor‐Net method in the program SplitsTree 4.14.6 (Huson, 1998; Huson & Bryant, 2006). Second, the split system is used in a two‐fold cross‐validation procedure via a random split method in the scikit‐learn library.

With this approach, we generated two SNP assignment sets: (1) North versus South, as defined by our sPCA results; and (2) Six regional groups, as defined by our DAPC results (Figure S2).

## 3 RESULTS

### 3.1 SNP genotyping and analysis

We obtained 524 million reads for 480 individuals (~1 M per individual) from GBS and identified 664,178 variants with the fastGBS pipeline. After variant filtering (details in Tables S1 and S2), we retained 6102 SNPs and 365 individuals (359 from China and six from South Korea).

#### 3.1.1 Population genetic diversity

*H_o_* in the Chinese populations ranged from 0.222 ± 0.003 to 0.306 ± 0.002, with the highest *H_o_* in populations around ~35–40°N and the lowest in Northeast China. This contrasts with the low heterozygosity we observed in our Korean samples, Kangwon (KOR, *H_o_* = 0.019), and Pocheon (KNA, *H_o_* = 0.021 ± 0.001) (Table 1). However, 31 Korean individuals in the populations Kangwon (KOR) and Pocheon (KNA) were identified as full siblings by SNPRelate. The *H_o_* was lower among most Korean samples compared to Chinese ALB (Figure S3). Expected heterozygosity and nucleotide diversity in Chinese populations showed patterns similar to *H_o_* with peak values ~35–40°N and the lowest values in the northeast. Similarly, *H_e_* and nucleotide diversity values in Kangwon (KOR) and Pocheon (KNA) were lower than for all Chinese populations (Table 1). Among Chinese samples, we observed significant isolation‐by‐distance (IBD, *R* = 0.56, *p* = 2.2e^−11^; Figure S4A) with clear latitudinal and longitudinal patterns among sampling sites (*H_o_*/*H_e_*, Figure S4B–E). Both *H_o_* and *H_e_* were negatively correlated with longitude, with heterozygosity decreasing from west to east, although these relationships were insignificant. Northern populations showed low *H_o_* and *H_e_*, but values increased with decreasing latitude until ~37°N, then dropped again for the southernmost site (Figure S4B,D). However, this relationship appeared to be driven by a single population, Cixi (CIX), and when we removed this population from the analysis, the latitudinal cline was no longer significant (Figure S5).

**TABLE 1:** Asian longhorned beetle sampling and basic population genetic analyses using 6102 SNPs, 16 locations in China, and 4 locations in Korea

ID	Locality	Country/Region	Latitude	Longitude	*N*	*H_o_*	*H_e_*	π
HRB	Harbin, Heilongjiang	Northeast China	45.8	126.54	21	0.226	0.233	0.24
CHC	Changchun, Jilin	Northeast China	43.82	125.32	10	0.222	0.223	0.238
TOL	Tongliao, Inner Mongolia	Northeast China	43.65	122.24	58	0.228	0.242	0.244
YJ	Yanji, Jilin	Northeast China	42.66	129.44	25	0.263	0.282	0.288
SHY	Shenyang, Liaoning	Northeast China	42.05	123.36	33	0.244	0.251	0.256
QI	Qingtongxia, Ningxia	Northwest China	38.02	106.08	28	0.297	0.302	0.308
YC	Yanchi, Ningxia	Northwest China	37.75	107.4	24	0.3	0.301	0.307
CHE	Chengde, Hebei	North China	40.98	117.95	17	0.283	0.274	0.284
IMC	Huhhot, Inner Mongolia	North China	40.83	111.66	21	0.261	0.256	0.262
BJ	Beijing	North China	39.75	116.14	10	0.284	0.281	0.296
SHI	Shijiazhuang, Hebei	North China	38.05	114.51	22	0.306	0.302	0.309
HS	Hengshui, Hebei	North China	37.74	115.67	20	0.299	0.303	0.311
JI	Jinan, Shandong	North China	36.65	117.12	12	0.278	0.279	0.293
TA	Taian, Shandong	North China	36.2	117.09	11	0.284	0.295	0.309
BB	Bengbu, Anhui	South China	32.92	117.39	20	0.297	0.301	0.308
CIX	Cixi, Zhejiang	South China	30.17	121.27	27	0.23	0.246	0.251
KOR	Kangwon	Korea	38.11	128.46	29	0.019	0.015	0.015
KNA	Pocheon	Korea	37.75	127.17	5	0.021	0.016	0.018
INC	Incheon	Korea	37.45	126.7	2	–	–	–
ULS	Ulsan	Korea	35.54	129.31	1	–	–	–

Note

Indices are calculated based on within‐population sample sizes ≥5; *N*, the exact number of samples per population. *H_o_*_,_ observed heterozygosity; *H_e_*, expected heterozygosity; π, nucleotide diversity. For further sampling details, see Table S1; for sampling and basic population genetic analyses involving microsatellites, see Table S3.

#### 3.1.2 Population genetic structure

In the PCA analysis, we observed Chinese and Korean populations were distinct (three individuals from KOR and KNA, upper right quadrant, Figure 2b). However, the three additional Korean samples from INC and ULS, collected in urban areas, were nested within the cluster containing samples from China.

**FIGURE 3:**
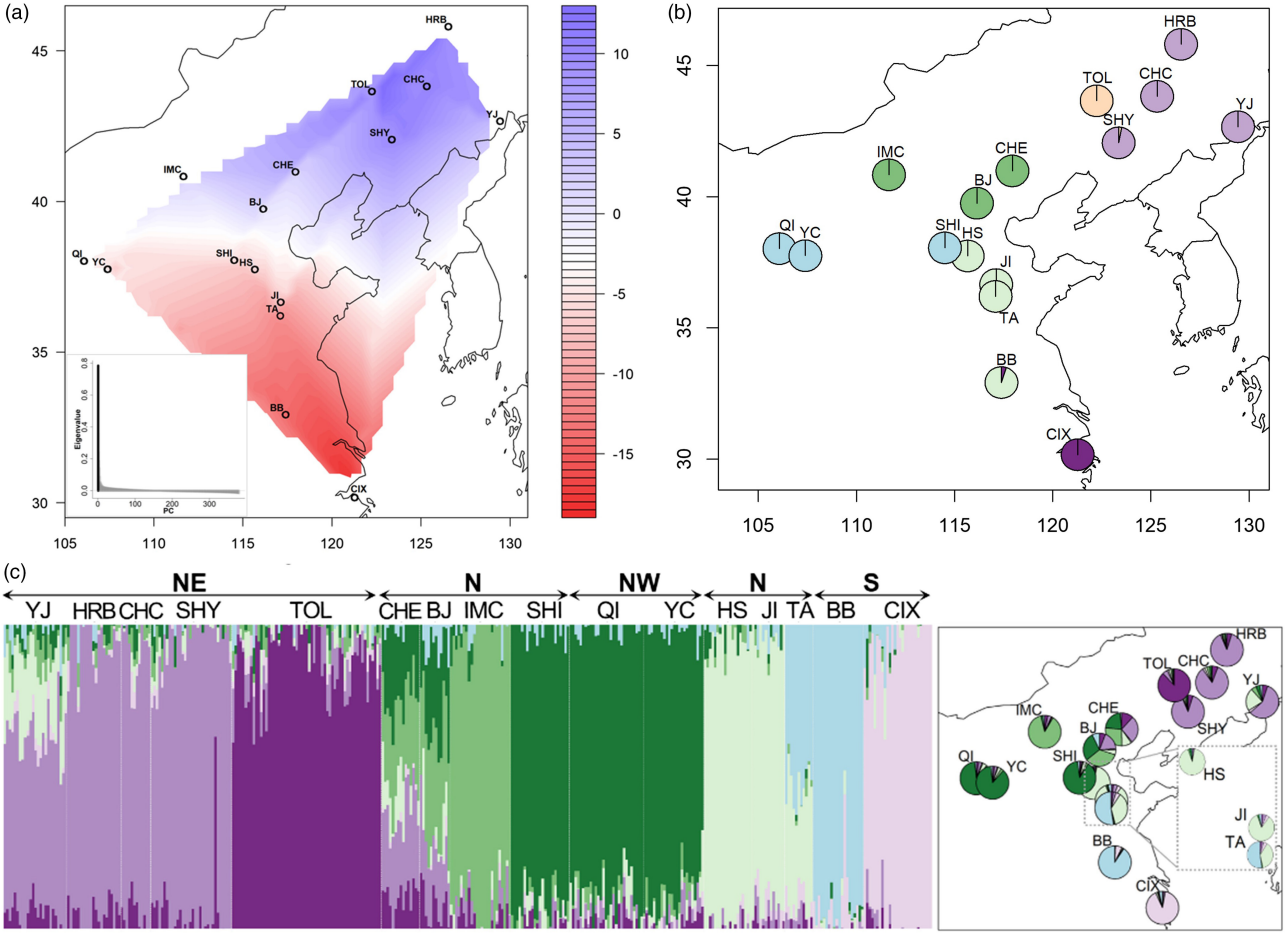
(a) Interpolated gradient map of the spatial genetic structure of the Asian longhorned beetle based on the first positive eigenvalue of spatial principal component analysis (sPCA). Eigenvalues of sPCA are displayed on the inset. Positive eigenvalues correspond to global genetic structure, and negative eigenvalues indicate local genetic structure. The color ramp shows values for lagged scores. (b) DAPC analysis using SNPs of 16 sampled ALB populations in China. Map with the 16 populations color‐coded as per the DAPC population clustering results. (c) Bar plot and interpolated map of ancestry coefficients across 16 populations (*k* = 7). A total of 6102 SNPs were used in the analysis. Each color‐coded pie represents one of the 16 sampled ALB populations in China. Population codes are explained in Table 1. A total of 6102 SNPs were used

Chinese ALB populations in the NW, S, and NE regions formed distinct clusters, while populations from the N region separated into three clusters, one that was unique, and the others were split among the NE and NW regions (Figure 2c,d; Figure S6). The sPCA analysis exposed a distinct north‐south genetic break (*p*‐value = 0.001) (Figure 3a), which was consistent with the *F*_ST_ values, where populations in the north were all highly divergent from those in the south (Table S5). DAPC analysis (Figure 3b) further resolved distinct subclusters among ALB populations within the northern and southern regions, with two distinct populations in the Northeast (SHY/CHC/HRB/YJ and TOL), one in North China (IMC/BJ/CHE), one in Northwestern China (QI/YC/SHI), and two along the eastern coast (HS/JI/TA/BB and CIX). We used these DAPC groupings in our AMOVA (i.e., six DAPC groups; refer to Figure S7A for BIC) and identified significant among‐group (14.8%) and among‐population within‐group (7.9%) genetic covariances (Table S6; *p* < 0.05).

Individual admixture estimates identified eight distinct populations (Figure 3c) based on the cross‐entropy criterion (Figure S7B). Admixture analyses showed that Chengde (CHE) and Beijing (BJ) (N region), as well as Yanji (YJ) (NE region), have the most complex admixture patterns, a result supported by relatively low *F*_ST_ values for these populations (Table S5). We also show that Yanchi (YC) and Qingtongxia (QI) (NW region), and Shijiazhuang (SHI) (N region) show similar genetic composition, further supporting our PCA, DAPC, and F_ST_ results. Finally, we observed similar genetic composition among Harbin (HRB), Changchun (CHC), and Shenyang (SHY) populations (NE region).

**FIGURE 4:**
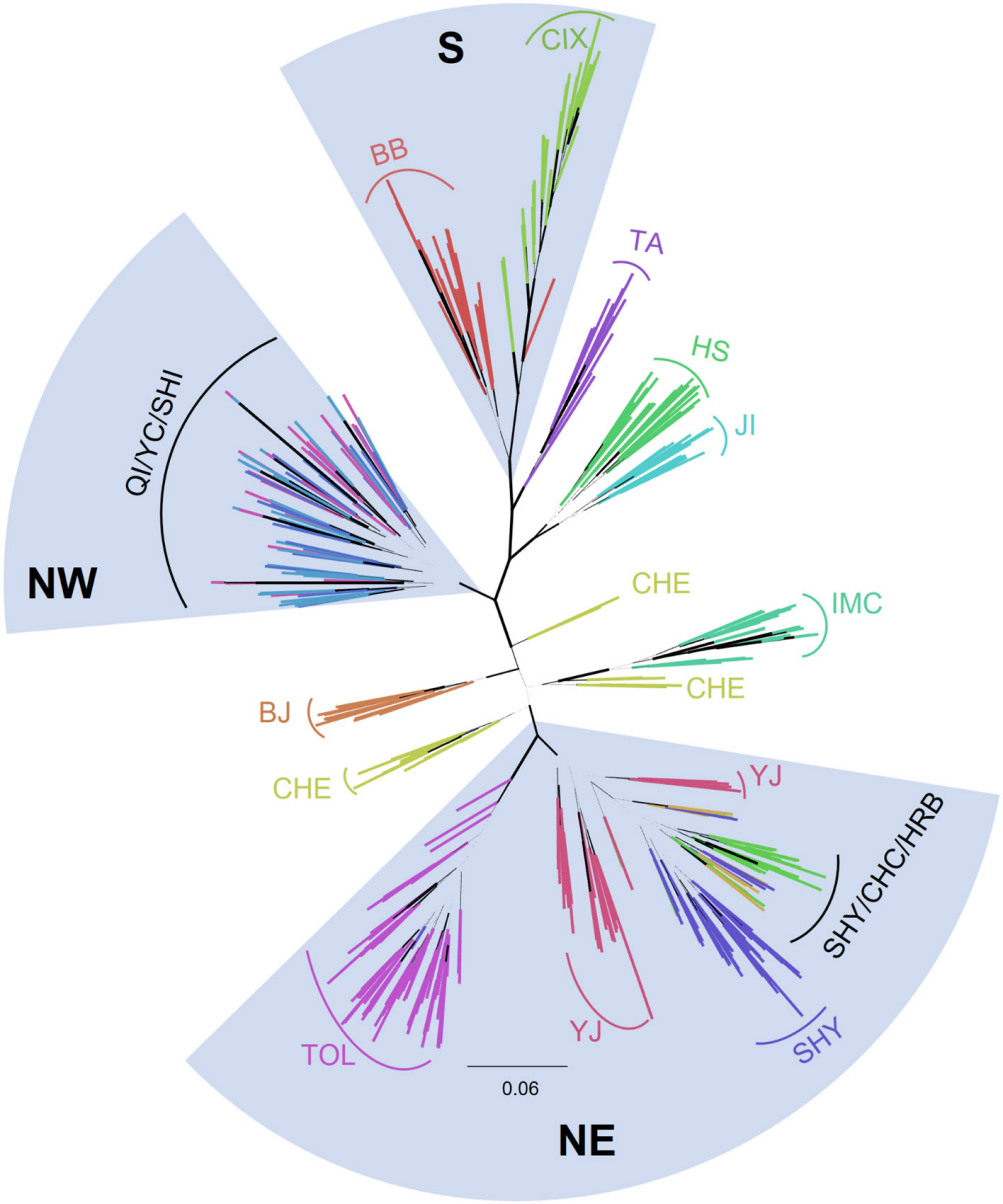
Maximum likelihood phylogenetic tree for Chinese Asian longhorned beetles analyzed in RAxML. A total of 6102 SNPs were used in the analysis. Each branch represents a sample. The branches are colored by their population codes (explained in Table 1). The width of each branch corresponds with their bootstrap value (widest branch with a bootstrap value of 100). Fan‐like sections indicate subregions NE, S, and NW (whereby SHI is actually in the N region). The rest of the populations are from the N region

The ML phylogenetic tree (Figure 4) revealed that subregions NW, NE, and S formed distinct, well‐supported lineages. However, the N region was polyphyletic relative to the other subregions. Individual populations formed distinct, well‐supported lineages with a few exceptions. Within the NW, individuals from Qingtongxia (QI), Yanchi (YC), and individuals from Shijiazhuang (SHI, from the N region) formed a single cluster. Both Chengde (CHE) and Yanji (YJ) did not form single lineages; instead, these populations were divided into several distinct clades. In the S subregion, the Bengbu (BB) population formed a distinct lineage, except for a single individual which grouped with Cixi (CIX), a population located further to the south (Figure 4, see also Figure 2a).

#### 3.1.3 Gene flow and population history

**FIGURE 5:**
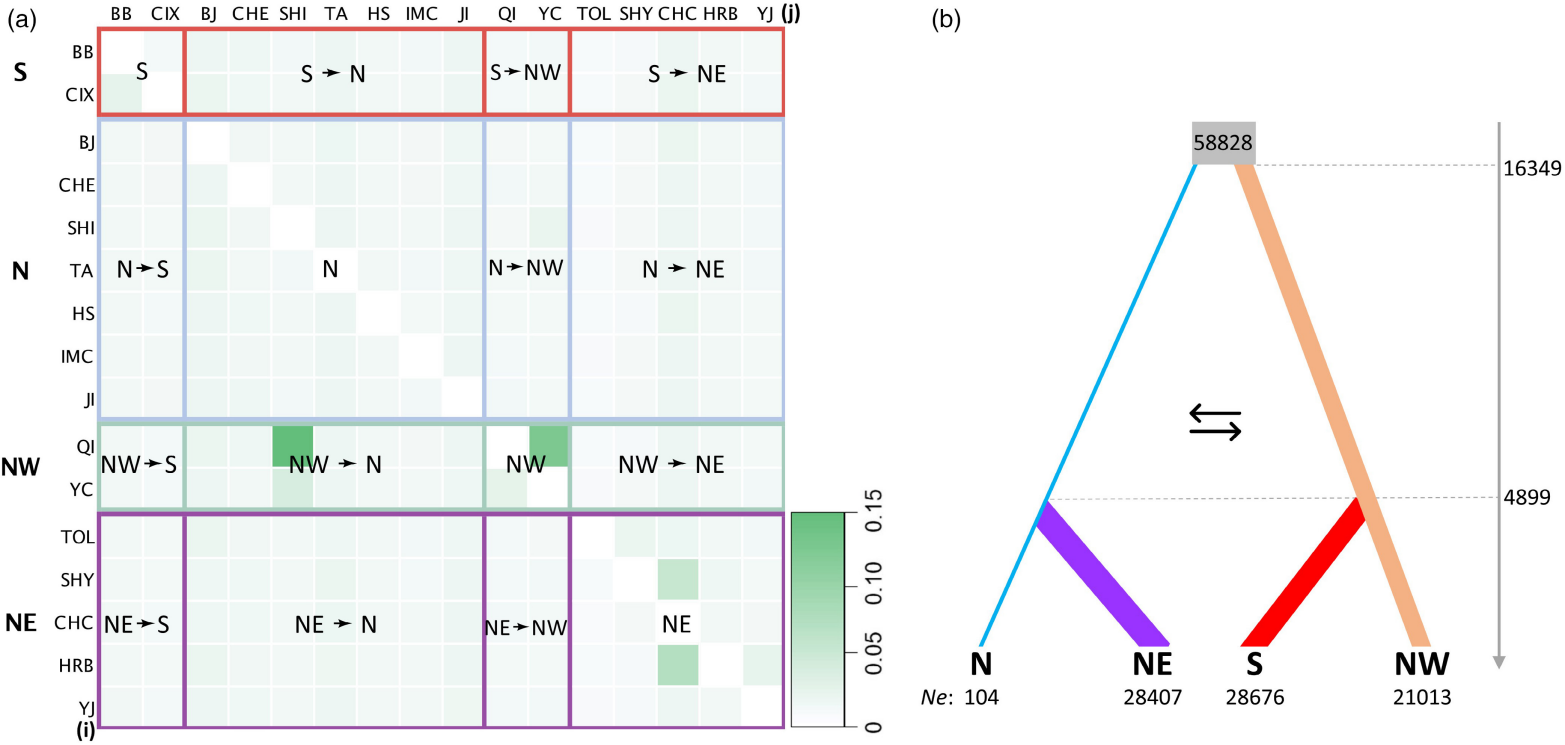
(a) Migration rates among the 16 Asian longhorned beetle populations sampled throughout China. The migration direction reads from populations in vertical (i) to populations in horizontal (j) order. Light to dark green color represents the migration rates from low to high. Contemporary gene flow within and between S, N, NW, and NE groups are indicated. Gene flow within each population is shown along the diagonal with empty values. A total of 6102 SNPs were used in the analysis. Population codes are explained in Table 1. (b) Population history model. The ancestral population is shown in gray. The estimated population divergence time (vertical axis) and effective population sizes (*Ne* values) are indicated in the figure. The width of each column reflects the relative effective population size

We estimated contemporary gene flow within and among regions using our SNP dataset. The migration rates ranged from 0.004 ± 0.004 to 0.124 ± 0.060 migrants per generation (Nm), with an average across all populations estimate of 0.01 ± 0.0006 Nm (Figure 5a). We see moderate gene flow among sites within the NW (0.104 ± 0.046 Nm) and NE (Shenyang (SHY)→Changchun (CHC) (0.043 ± 0.032 Nm) and Harbin (HRB)→Changchun (0.058 ± 0.032 Nm)). The highest rates of gene flow occurred between sites in the NW (Qingtongxia (QI) and Yanchi (YC)) to Shijiazhuang (SHI) in the N (0.124 ± 0.060 and 0.031 ± 0.019 Nm, respectively). The trace plot and probability distribution are shown in Figure S8.

Out of the nine scenarios that included present‐day populations tested in Migrate (Figure S1), model3 was the most probable based on marginal likelihood (Table S7). This model assumes that the North population first diverged from the Northwest population with ongoing gene flow and an estimated migration rate of 5e^−03^ between populations (Fastsimcoal output). Through modeling by Fastsimcoal, the divergence between the North and the Northwest populations was traced back to 16,349 generations ago (Figure 5b). Furthermore, the South population diverged from the Northwest population, and the Northeast population diverged from the North population (see Figure 2a for delineation of regions). The respective splits of the Northeast and the South populations were estimated to have occurred 4899 generations ago (Figure 5b). The highest effective population size (*N_e_*) was predicted within the hypothetical ancestral population (*N_e_* = 58,828 ± 4449), with a moderate reduction in *N_e_* following divergence of the NE (28,407 ± 4711), NW (21,013 ± 4509) and S (28,676 ± 4,749) descendant populations. The population of ALB in the North exhibited surprisingly low *N_e_* (104 ± 4796) (Table S8).

#### 3.1.4 Selection detection

**FIGURE 6:**
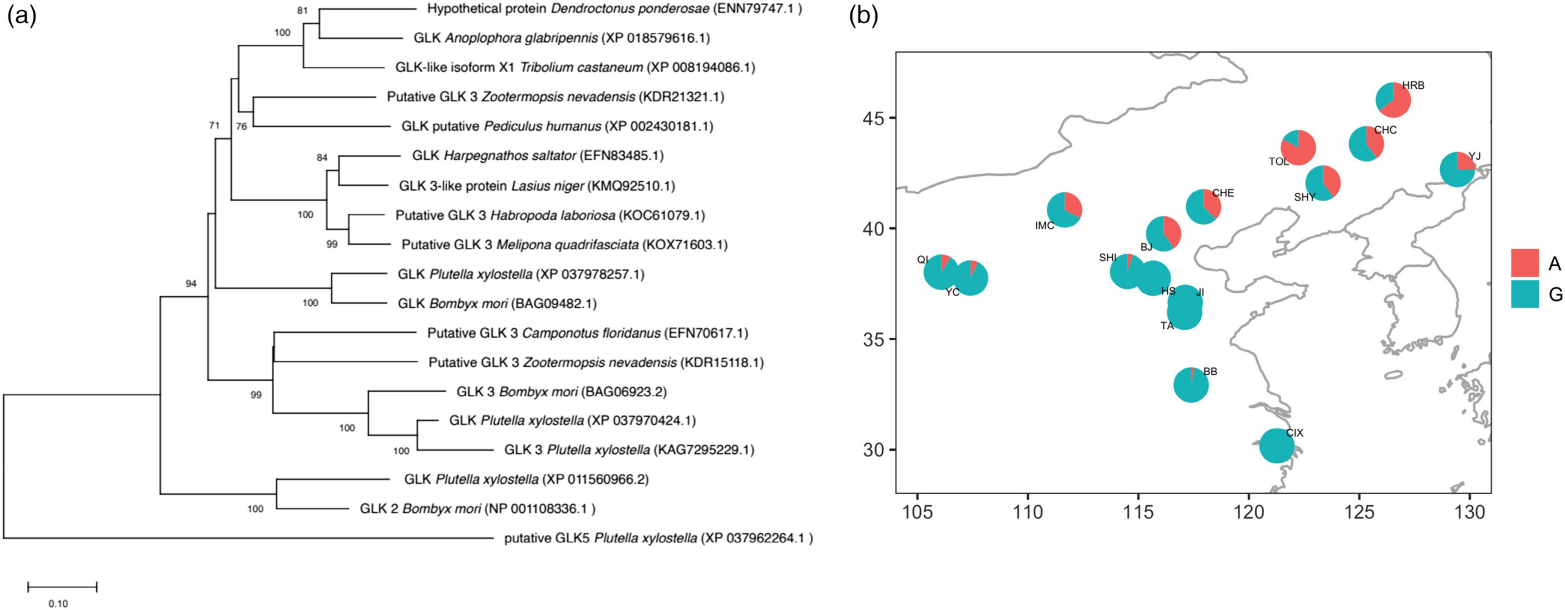
Exploring the potential functional relevance of a missense mutation under positive selection (*F*_ST_ = 0.335) within the *Anoplophora glabripennis* glycerol kinase gene *AGLA000593*. (a) Phylogenetic tree of several glycerol kinase proteins from different insect species, including *A*. *glabripennis*. All NCBI accessions are provided. The neighbor‐joining tree was done in MEGAX (with 1000 bootstraps). Only the branch supports above 70% are indicated. The closest homology for the ALB protein was found with *D*. *ponderosae*. (b) Geographic map of the allele frequency distribution for the glycerol kinase gene *AGLA000593* across the 16 Chinese ALB populations studied by GBS technology. G refers to the reference allele and A to the alternative allele (the missense mutation)

We did not detect outliers in OutFLANK using a q‐value cutoff of 0.05, while we did detect SNP loci putatively under selection through *fsthet* (Figure S9; Table S9). Among these 360 outliers (48% under positive selection; 52% under balancing selection), there were 185 (51%) intergenic variants, and 172 variants (48%) were annotated as related to protein coding (of which 29% were intron variants; 37% in upstream or downstream regulatory regions; 34% directly in the coding DNA sequence CDS). Among the *F*_ST_ outliers directly associated with genes, we found SNPs within 134 genes. For 26 genes, multiple outlier loci were detected (i.e., two to four within a single gene). Of those 58 SNP outliers directly within the CDS, there were 31 synonymous variants and 26 missense variants, and one splice variant. We assigned functional annotations to each locus based on flanking sequence similarity with previously published results. Over half of the flanking sequences (*n* = 213) could not be characterized. For loci that were functionally similar to previously annotated regions, we identified loci associated with a range of molecular functions and biological processes (Table S9). Of particular interest was the glycerol kinase (*GLK*) gene (AGLA000593, i5K database, https://i5k.nal.usda.gov/Anoplophora_glabripennis, Accessed Jan 2022). The SNP under selection was annotated as a missense mutation under positive selection (*F*_ST_ = 0.335) (Table S9). We compared the ALB *GLK* gene to previously published insect *GLK* genes. We observed that the ALB *GLK* gene is closely related to genes described in other beetle taxa (Figure 6a), including *Dendroctonus ponderosae* and *Tribolium castaneum*, in agreement with the species’ phylogenetic relationships shown in McKenna et al. (2016). We plotted the allele frequency of the SNP (A/G) and showed that the allele frequency exhibits a distinct clinal trend within our ALB populations (Figure 6b; for allele count details, see Table S10).

### 3.2 Microsatellites analyses

After correcting for sample size variation, allelic richness is similar across locations (Table S3). A slight deficiency of heterozygotes was observed in all populations (all highly significant, *p* < 0.01). Linkage disequilibrium was minimal among those 53 microsatellite loci, as only six out of all 1378 (0.44%) pairwise comparisons had significant false discovery rate‐adjusted *p*‐values. LD was also checked for within each population. There were no loci pairs found showing LD in every population, and the maximum number of populations that shared an LD loci pair was seven, which was less than half of the total number of populations.

**FIGURE 7:**
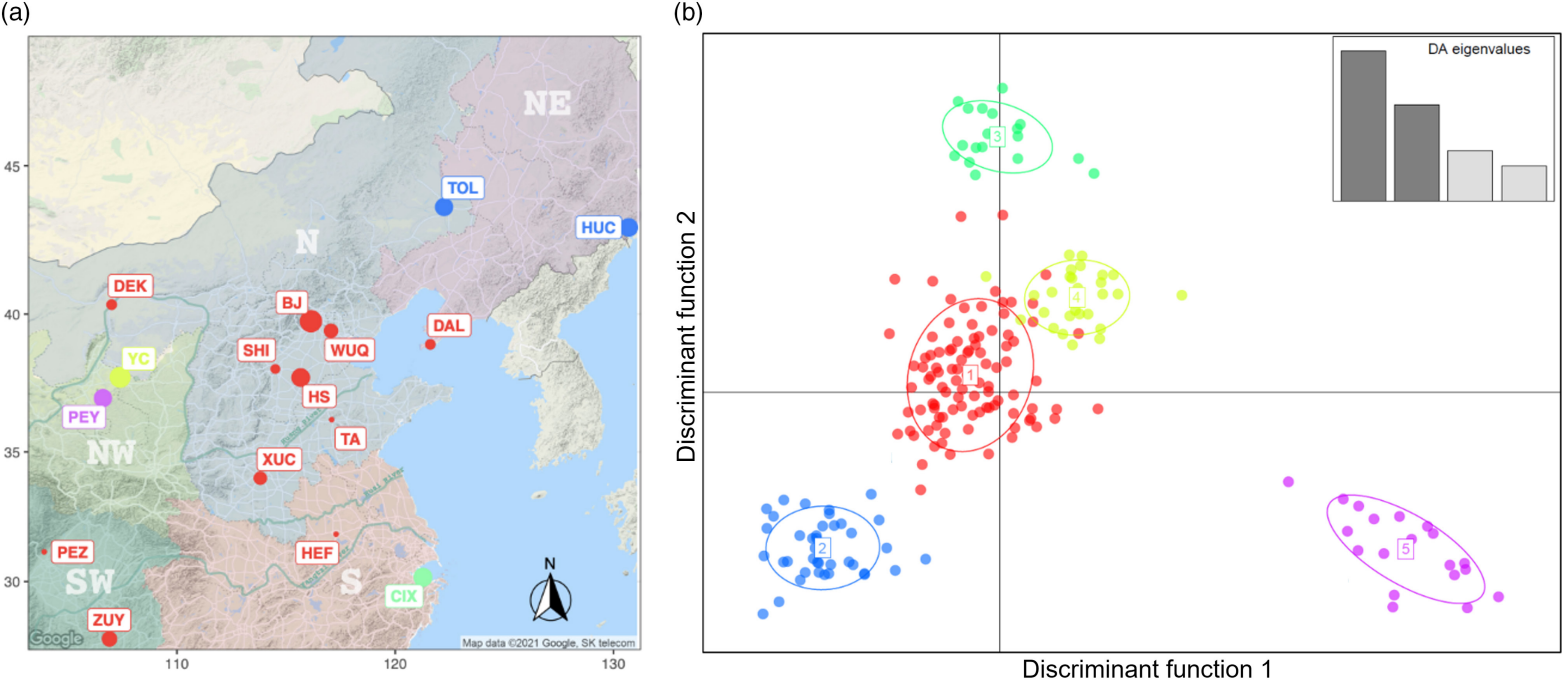
Discriminant analysis of principal components (DAPC) of the Asian longhorned beetle using microsatellites. ALB sampling for microsatellites analysis covers different regions in China. (a) Sampling locality. The color for each locality corresponds to its cluster in DAPC. The bubble size of each population is relative to the sample size. The codes for the populations are shown in Table S3. (b) Five clusters were identified in DAPC

The Ward algorithm did not determine a single optimum number of genetic clusters and found approximately equal support for models from a range of *K* = 4 to 6 (24 PCs retained), with *K* = 5 having the lowest Bayesian Information Criterion (BIC) score (Figure S7C). When the native populations were assigned into five genetic clusters, cluster 1, the largest, was formed mostly by specimens from North China. Three populations outside North China, Dalian (DAL), Pengzhou (PEZ), and Zunyi (ZUY), were genetically embedded in cluster 1 (Figure 7). The NE populations Tongliao (TOL) and HuiChun (HUC) (cluster 2) and the eastern coastal population Cixi (CIX, cluster 3) can clearly be distinguished from cluster 1 on the second discriminant function. Two NW populations Yanchi (YC) and Pengyang (PEY), merely 200 kilometers apart, were very distinct, forming the separate clusters 4 and 5, respectively. For the model with *K* = 4, Yanchi (YC) merged with cluster 1. And if clustering was increased to K = 6, an additional cluster was formed by seven specimens from Zunyi (ZUY) that became separated from cluster 1. However, another five specimens collected in the same location remained with cluster 1. For the AMOVA with *K* = 5, most of the genetic variance (84.56%, *p* < 0.001) is found within locations, with 7.22% (*p* < 0.001) of the variance among locations within clusters, and 8.22% (*p* < 0.001) of the total variance among clusters.

### 3.3 Congruence of genetic markers

We used a Mantel test between the two *F*_ST_ matrices generated from SNPs and microsatellites to assess congruence between our marker and specimen sets (Tables S5 and S11). Here, we observed a significant congruence between our datasets (*r* = 0.951, *p*‐value = 0.039) based on 9999 replicates between the two datasets. We observed similar, although not identical, clustering in our two DAPC analyses on these distinct datasets (Figures 3b and 7). Furthermore, we also found that the positions of the N‐S break between regions in the two sPCA analyses were the same (Figure 3a; Figure S10).

### 3.4 Population assignment using SNP markers

**FIGURE 8:**
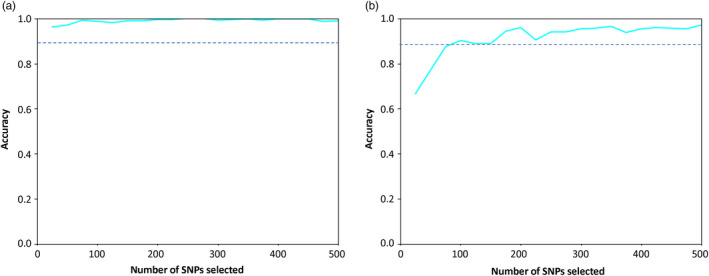
Prediction accuracy of Asian longhorned beetle individual assignment with an increasing number of SNPs selected (ranging from 20 to 500). (a) ALBs were assigned to two groups as identified in sPCA. (b) ALBs were assigned to six groups identified in DAPC. Dashed line indicates 90% accuracy of assignment

The accuracy of assignments to different groups was conducted using an increasing SNP set (up to 500 SNPs; Figure 8). We assigned individuals to the N and S groups with >90% accuracy using only 20 SNPs that we ranked as the most discriminant and with near 100% with 100–500 SNPs (Figure 8a). Assigning individuals to one of the six DAPC groups reduced accuracy to 96.4% with 200 SNPs (Figure 8b). Assignment accuracy for each population assignment class using 500 SNPs was shown in Figure S11. When the whole set (6102 SNPs) was used in the tests, accuracies were 100% (two groups) and 98.6% (six groups), respectively.

## 4 DISCUSSION

The Asian longhorned beetle is a widespread pest found throughout the temperate forests in East Asia. Global spread of this species underlies the urgent need for biosurveillance tools that can trace its pathways of invasion and monitor its invasion dynamics. However, such tools require detailed knowledge of the population structure of ALB in its native range. Here, our results show pronounced regional differences, indicative of historical biogeographic structuring, as well as evidence of contemporary migration between China and South Korea and within regions in China. We also identified several genomic regions under selection and that may underlie adaptive differences between populations. Our data also provide a foundation for future genomic‐based surveillance tools. To that end, we identified smaller sets of SNPs that accurately assigned individuals to regions and subregions within the native range. We further highlight how these markers can be used to assign individuals to regions within the native range.

East Asia has a complex biogeographic history. Temperate forests and their associated communities experienced repeated fragmentation and range shifts due to historic changes in climate and sea levels, combined with diverse topography in the region. These biogeographic processes created ideal conditions for the evolution of high inter‐ and intraspecific diversity (Bai et al., 2010; Chen & Lou, 2019), which can result, at least partially, from the biogeographic history of the area. We observed some distinct phylogeographic patterns within ALB consistent with earlier publications and patterns seen in other forest species (Carter et al., 2009; Du et al., 2019, 2021). First, we observed a distinct cluster of Korean ALB specimens from forested areas in Kangwon (KOR) and Pocheon (KNA) (except three individuals sampled from urban areas, which will be discussed further below). These populations were distinct from ALB populations in China, consistent with previously published results (Carter et al., 2010; Javal et al., 2019a; Lee et al., 2020) and were collected in the native forest from the northeast region of the country, within the hypothesized historic distribution of the species (Lee et al., 2020). Our limited sample of insects from this forested region showed low genetic diversity, contrary to the more extensive sampling in Lee et al. (2020). Given that our samples from Kangwon (KOR) were collected from a few trees at a rest stop in Korea and proved to be highly related based on kinship estimation performed in SNPRelate (Table S12), these can, therefore, be considered isolated populations with low genetic diversity due to inbreeding and sampling bias. Despite these differences, the unique Korean population structure and genetic diversity we observed in ALB, relative to mainland China, were consistent with phylogeographic patterns observed in other forest species (reviewed in Qiu et al., 2011), including assassin bugs (Du et al., 2019), raccoon dogs (Kim et al., 2013), orchids (Tian et al., 2018), and oaks (Zeng et al., 2015). Baekdudaegan, the main mountain range that runs the length of the Korean Peninsula, has been identified as an important biodiversity hot spot that served as a glacial refugium for boreal and temperate forest species during the last glacial maximum (LGM) (Chung et al., 2018). Sea level changes created and flooded land bridges between the Korean Peninsula and mainland China, isolating species with previously continuous distributions (Qiu et al., 2011). Consequently, ALB populations in this forested region of Korea are genetically differentiated from mainland China through evolutionary processes.

Complex phylogeographic structure is also typical among forest species in eastern China (Qiu et al., 2011). We found that ALB genetic variation was hierarchically structured, with sharp regional genetic breaks and internal population subdivisions with varying levels of admixture. Regionally, ALB populations were divided into northern and southern populations, similar to, but more clearly defined than, results in earlier publications (Carter et al., 2009; Javal et al., 2019a). This North‐South population break is seen in a wide range of forest species (reviewed in Qiu et al., 2011), including ALB host plants (*Betula*, Chen & Lou, 2019; *Acer*, Guo et al., 2014; *Populus*, Hou et al., 2018). In our SNP dataset, this North‐South division aligned with the hypothesized location of an “aridity belt” along the Yellow River (= Huang He) in East Asia (Milne & Abbott, 2002). Geographic barriers such as mountain chains, arid regions, rivers, and geographic distance are key factors driving population structure in many widely distributed Asian insect species (e.g., Du et al., 2021).

Population structure can be driven by a combination of geographic, spatial, and environmental conditions. Often, these vary along spatial or temporal gradients; temperature conditions, light regime, and moisture availability can show latitudinal, altitudinal, or longitudinal gradients across a species range (De Frenne et al., 2013; Laiolo & Obeso, 2017). Geographic barriers such as rivers and mountains, particularly when aligned along these longitudinal or latitudinal axes, can profoundly influence population structuring and gene flow within populations (Bester‐van der Merwe et al., 2011). We observed latitudinal gradients in ALB with genetic diversity peaking between 35°N to 40°N (Figure S4), similar to previous findings in ALB (Javal et al., 2019a), and spotted lanternfly (Du et al., 2021; Zhang et al., 2019). Thus, the higher genetic diversity found for the central populations compared to the edge populations would support the “center‐periphery hypothesis” for ALB’s native distribution.

Climate is critical in shaping biogeography within continents (Ficetola et al., 2017; Mckown et al., 2014). For ectothermic species such as insects, temperature is particularly influential in defining species ranges (Angilletta, 2009). Surviving subzero temperatures is physiologically challenging and can limit the northward expansion of insects (Brightwell et al., 2010). This hypothesis aligns well with our results, where we observed that the genetic diversity in ALB is negatively correlated with latitude. A notable exception represents the Cixi (CIX) ALB population (30°N) which shows relatively low genetic diversity. Contrary to the other population sampled in the South (Bengbu, 33°N), Cixi is situated south of the Huai River, thus already within the subtropical region with winter temperatures above 0°C. While ALB is found throughout East China, it is generally considered a temperate species (Feng et al., 2016b; Javal et al., 2018; Keena & Moore, 2010), and the greatest population density is north of the 0ºC isotherm (Yan, 1985). Therefore, although ALB has been found throughout China, their populations may be limited by biotic pressures (Fragnière et al., 2015), hence, limiting their effective population size and leading to the lower genetic diversity observed in the southernmost population CIX, and the northernmost populations HRB and CHC in terms of latitude.

### 4.1 Population history and contemporary movement

The relative contribution of contemporary dispersal and historic population structure represent two opposing forces when seeking to reconstruct the population structure of an organism. Contemporary movement is thought to blur the historic population boundaries that evolved over thousands of years. Contemporary dispersal, as influenced by anthropogenic movement of ALB throughout the native range, was hypothesized to have significantly disrupted the natural population boundaries (Carter et al., 2009; Javal et al., 2019a). We detected evidence of gene flow and admixture within the native range. Globally, gene flow was low between regions, except for specific sites in the NW and N contributing to gene flow between the two regions. Our population history modeling dated evidence to c. 16,300 years ago in our most probable demographic scenario. Indeed, such dispersal for ALB might have been driven by changing environmental factors coinciding with the retreat of the glacial coverage that began c. 18,000 years ago as the end of the LGM. It is probable that ALB experienced similar fragmentation and distribution restrictions as its host plants, allowing congruent phylogeographic structuring to evolve. Then, this regional population structure would have been maintained given ALB’s low dispersal ability (Smith et al., 2001, 2004; Williams et al., 2004) and limited movement beyond the natal tree. The second major population split and scattering of ALB was dated to c. 5000 years ago, thus following the Holocene climatic optimum in China (6000 years ago), when temperate deciduous forest vegetation reached almost 1000 km further north (48°N) than at present and extended further southwestwards at higher elevations than today (Yu et al., 2000). As climatic restrictions eased, ALB expanded its range in concert with its host plants, leading to localized admixture between nearby regions while still maintaining distinct regional structure. Similar population structuring, including regional admixture, has been reported in temperate forest trees, including *Juglans* species (Bai et al., 2016) and *Betula platyphylla* (Chen & Lou, 2019). We also observed evidence of movement out of the North to outlying regions in Zunyi (ZUY) and Pengzhou (PEZ) in the microsatellite dataset (Figure 7), suggesting a possible long‐distance spread pathway. Similarly, we detected two distinct genomic signatures in our South Korean samples. We had specimens collected from sites within the historic forested range of ALB (i.e., Kangwon, Pocheon), and three additional individuals collected within the urban centers of Incheon (INC) and Ulsan (ULS). Lee et al. (2020) suggests that ALBs in urban centers do not represent local populations but are the result of long‐distance dispersal from China. Our results support these conclusions: individuals in Incheon (INC) and Ulsan (ULS) nest within the Chinese samples, and group with Jinan (JI) and Taian (TA) in Shandong Province, respectively (Figure S12) (see also: Javal et al. (2019a); Lee et al. (2020)), with Shandong being an important shipping hub between China and South Korea (Li, 2012). As ALB are poor dispersers (Smith et al., 2001, 2004; Williams et al., 2004), the current long‐distance movement is likely linked to human activities. Although our findings do not support the hypothesis that contemporary anthropogenic movement was widespread and blurred regional population differences (Carter et al., 2009; Javal et al., 2019, 2019,2019, 2019), we were, nonetheless, able to detect cryptic movement of ALB within the native range.

### 4.2 Selection detection

We identified several loci under selection within our ALB populations. We were particularly interested in glycerol kinase (GLK), an enzyme that catalyzes an important rate‐limiting step in the utilization of glycerol at diapause termination (Kihara et al., 2009). Glycerol is composed of two polyols and is a well‐known cryoprotectant molecule used by insects to prevent intracellular ice formation (e.g., Cheng et al., 2014; Park & Kim, 2013; Storey & Storey, 2012). Prior evidence also showed that ALB exhibits both seasonal and population differences in the accumulation of this important cryoprotectant (Feng et al., 2014, 2016), which coincide with gene expression that peaks during winter (Xu et al., 2021). Moreover, recent laboratory experiments performed on ALB colonies showed that glycerol content changes within the hemolymph occur during diapause and that such changes are related to concurrent shifts in the supercooling point in ALB (Torson et al., 2021). In our study, we found that the missense mutation within the *GLK* gene occurs at much higher frequencies at higher latitudes and follows a clear clinal trend. Based on the observed allele frequency pattern, it suggests that the northern allele variant may provide an adaptive advantage to the northern populations, and further study of the functional implications of this missense variant would be fruitful. We also suggest that this locus be prioritized in future genomic biosurveillance panels (Roe et al., 2019), given its potential link with cold tolerance in ALB. However, accurate SNP calling requires a reference genome, which is not often available for nonmodel organisms. Moreover, functional genomics research would benefit from a properly annotated genome of the organism under study, especially for the gene space, and we note that over half of the protein coding loci could not be identified. Much of ALB’s genome annotation is still under development because its genome sequence is highly fragmented (McKenna et al., 2016), and an annotated chromosomal assembly would greatly improve detection of other loci of interest and adaptive differences among populations.

### 4.3 Dataset congruence

Here, we used two different genetic marker systems and slightly different sampling strategies within the main geographic regions to verify the overall genetic pattern of ALB in its native range. In addition to complementary findings, in both cases, the overall genetic structure differentiated NE, NW, and S regions. Given the highly congruent results from the two sets of independent markers, it is worthwhile to consider the pros and cons of the two approaches. The biggest advantage of GBS is undoubtedly its capability to produce thousands of SNPs. Genome‐wide SNP markers provide in‐depth insights into the level of population structure that may not be revealed by other types of genetic markers. For example, our SNP data show a genetic cline along a latitudinal gradient in Eastern China, a result not reported by Sanger sequencing or microsatellite data (Carter et al., 2010; Javal, Lombaert, et al., 2019). For organisms whose population structure is obscured by recent long‐distance dispersal (such as human‐aided movement as seen in many invasive species), SNP‐based analysis proves to be particularly powerful in resolving the underlying genetic pattern (Picq et al., 2018). Additionally, GBS can generate SNPs that are candidate markers for identifying associations between genotypes and phenotypes that may point to processes of selection or adaptation to novel environmental conditions (Wickland et al., 2017), which can ultimately improve our assessment of invasive risk.

However, microsatellite markers do have their own merits. Using the designed PCR primers, there is no need to sequence the genomes and run the bioinformatic pipeline again for joint SNP calling when new samples are added to the existing database. Although it is not always possible to compare different datasets produced by different laboratories due to inconsistencies in allele size calling (Vignal et al., 2002), combined with its proven capability to resolve population structures and less requirement on bioinformatics, microsatellite markers will continue playing an important role in population genetic studies.

### 4.4 Developing genomic tools for biosurveillance

Preventing the incursion of invasive forest pests is paramount to an effective biosurveillance program. One of the most critical applications in an effective biosurveillance program is (a) accurate detection of regulated pests to enable an early, rapid response; (b) ability to trace interceptions back to the source and inform trade regulations and policies (Bilodeau et al., 2019; Hamelin & Roe, 2020; Roe et al., 2019). Given the consequences of inaccurate information, it is imperative that the identification and assignment of intercepted invasive species is robust and accurate. As such, genomic tools are becoming the “gold standard” to support regulatory decision‐making (Bilodeau et al., 2019; Roe et al., 2019). Our genomic results showed equally strong genetic differentiation among native ALB populations, with limited contemporary migration. Therefore, we were able to exceed 90% predictive accuracy with only 200 SNP markers for the six genetic groups delimited within our dataset. We needed even fewer SNP markers to confidently assign individuals to Northern or Southern regions with 95% accuracy. A study on *Lymantria dispar* spp. also showed that a high degree of genetic differentiation led to high population assignment success (Picq et al., 2018). They used laboratory colonies that consisted of eight groups that were genetically distinct, and the assignment success was generally high (86%–100%) using different sets of SNPs (12, 24, 48, and 96). However, for populations of white bass (*Morone chrysops*) with a moderate genetic differentiation between six populations (*F*_ST_ = 0.083), the assignment accuracy reached 99.78% with 57 SNPs (Zhao et al., 2019b). Moreover, progress has been made in developing assignment tools using SNP markers. For example, an assay that contains 324 SNPs, allowing for predictions of phenotype, biogeographical ancestry, and male lineage has been demonstrated to be a robust and sensitive human forensics tool (Diepenbroek et al., 2020). Overall, such an approach is also very promising for translating our results into developing SNP panels for a biosurveillance purpose in forest protection. While our sampling design covered most of the regions in China, there are records of ALB throughout China (Yan, 1985), and we have not fully sampled the entire distribution. Given our microsatellite results, the NW population Pengyang (PEY) that is not included in the SNP dataset formed a distinct cluster. There are likely other populations with a different genetic make‐up that have not been collected. Therefore, it is necessary to consider the possibility of unsampled sources when moving forward with genomics‐based biosurveillance.

## 5 CONCLUSION

Our study revealed clear regional differentiation among native ALB populations using two independent datasets that comprised complementary genetic data and sample sets. Biogeography, drift, and limited dispersal capacity are likely key factors that aided the formation and subsequent maintenance of the ALB population structure within its native range. Future research is poised to explore the environmental factors, including both abiotic (e.g., temperature, precipitation, etc.) and biotic (e.g., host plants), that shaped the complex genetic structure we observed in this pest. The patterns of genetic structure varied among regions and while large‐scale human‐assisted ALB population migration was limited, we were able to detect it in both datasets. Our ability to resolve this complex pattern of movement in the native range demonstrated the ability for our markers set to serve as a diagnostic tool to track invasive ALB populations outside their native range. Using our genome‐wide markers in a diagnostic framework will lead to the development of biosurveillance tools to aid rapid screening and pathway analyses of new ALB interceptions in support of plant protection agencies and regulatory bodies.

## CONFLICT OF INTEREST

The authors declare no conflict of interest.

## Supporting information

Figure S1‐S12

Tables S1‐S12

## Data Availability

Raw sequence data are available at the Sequence Read Archive (SRA) with the BioProject ID PRJNA824548. Variant Call Format data have been deposited in the Dryad Digital Repository: https://doi.org/10.5061/dryad.866t1g1rb. Scripts are available at https://github.com/mimingcui/nativeALB.
