# Supplementary material for: Genome‐scale phylogeography resolves the native population structure of the Asian longhorned beetle, Anoplophora glabripennis (Motschulsky)
Source: Evol Appl. 2022 Jun 7;15(6):934–53. doi: 10.1111/eva.13381 (PMC9234632; doi:10.1111/eva.13381)
Supplement: Supplementary file 1 — Figure S1‐S12 [file EVA-15-934-s002.pdf]

Supplementary Figures

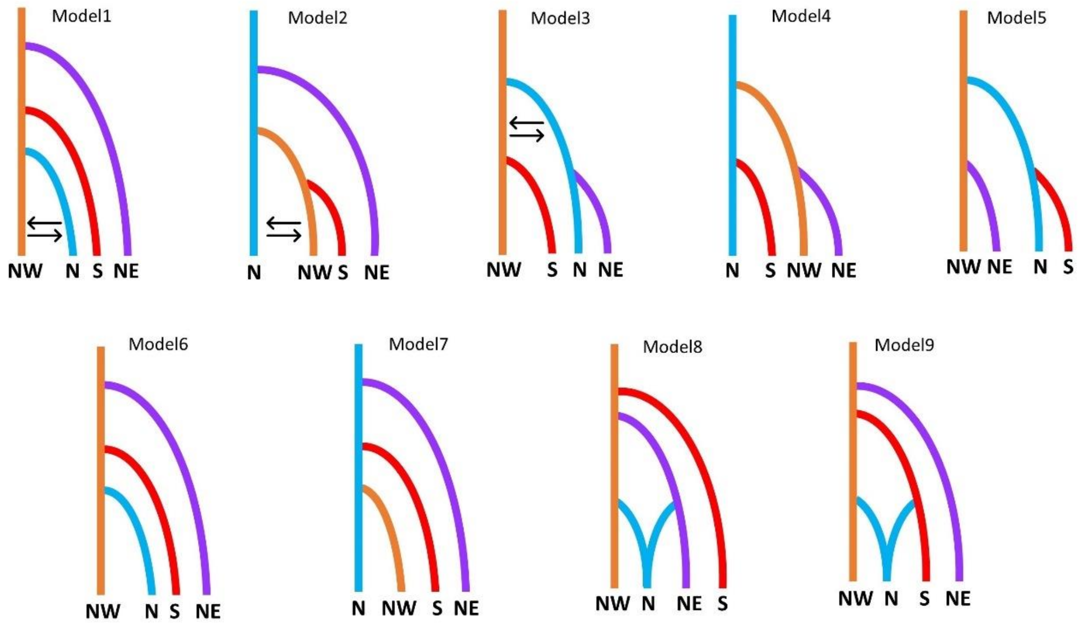

Figure S1 Nine demographic models tested in Migrate. 422 SNPs shared by 80 samples were used in the analysis of four groups identified by the PCA, *i.e.*, Northwest (NW, orange), North (N, turquoise), South (S, red) and Northeast (NE, purple). Gene-flow is indicated by arrows. The divergence times between groups were not tested here.

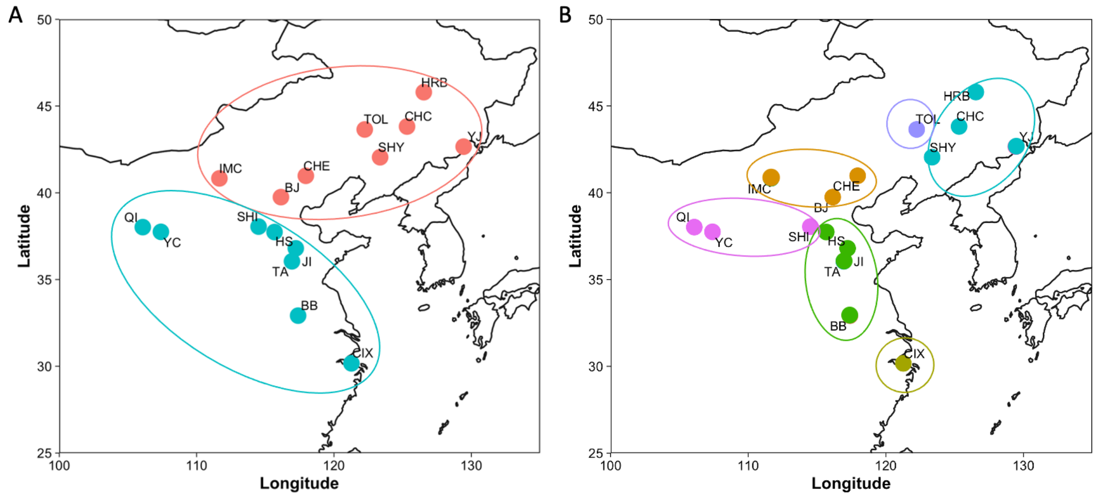

Figure S2 Grouping map. **A** Two groups based on sPCA results. **B** Six groups according to groups identified by DAPCs. For population codes, see Table 1.

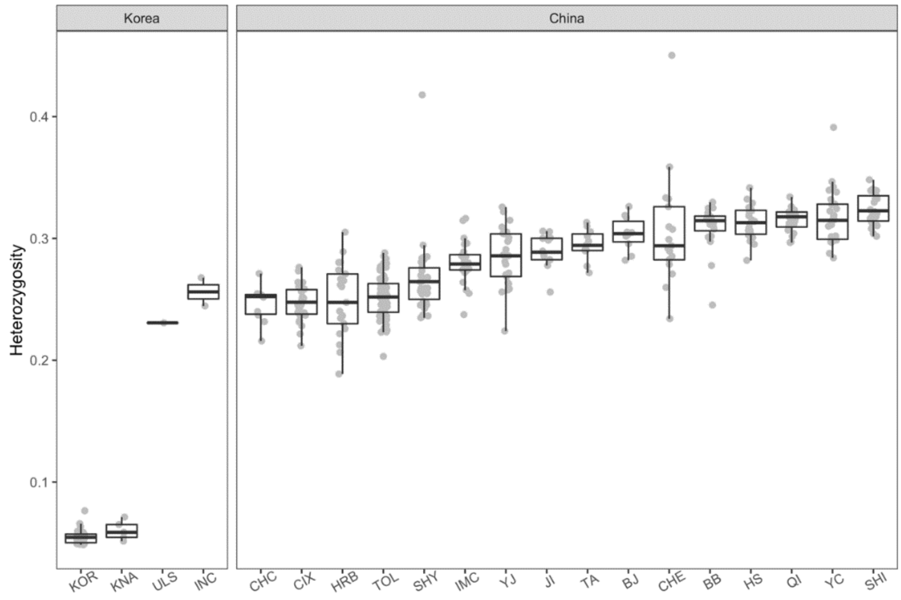

Figure S3 Observed heterozygosity plot for ALB individuals. Each dot represents an individual. The information for sampling site is shown in Table 1**.** Populations are ordered by mean values in ascending order. A total of 6,102 SNPs were used in the analysis.

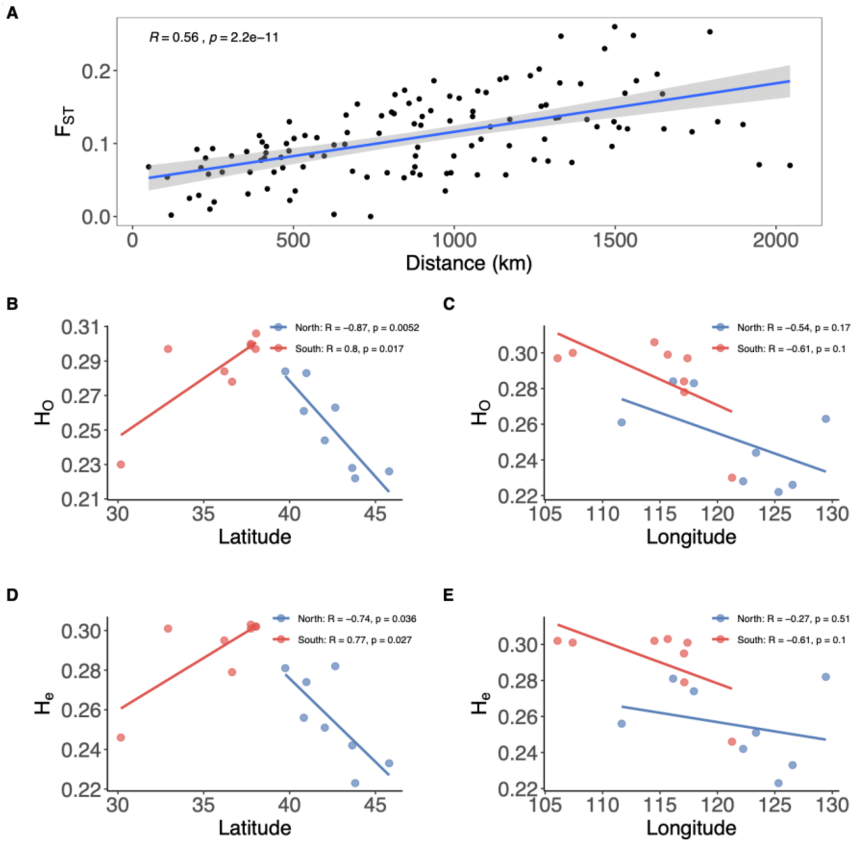

Figure S4 A Isolation-by-distance shown as pairwise F_{ST} between populations against geographical distances. **B**, **C** Relationships between observed heterozygosity and latitude/longitude. **D**, **E** Relationships between expected heterozygosity and latitude/longitude. The eight populations in the north identified by sPCA are colour-coded in blue and the other eight populations in the south in red. Colour-coded lines are the trend lines. Pearson correlation coefficient and *P*-value are presented on the top right of each graph.

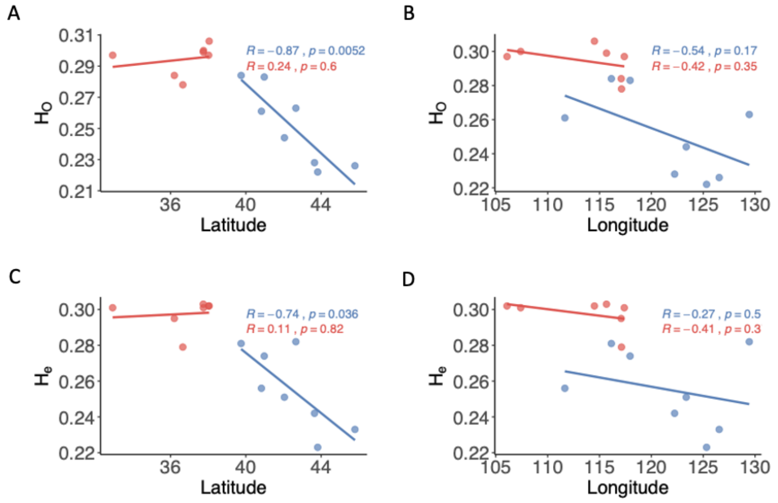

Figure S5 A, **B** Relationships between observed heterozygosity and latitude/longitude excluding population CIX. **C**, **D** Relationships between expected heterozygosity and latitude/longitude excluding CIX. The eight populations in the north identified by sPCA are colour-coded in blue and the other eight populations in the south in red. Colour-coded lines are the trend lines. Pearson correlation coefficient and *P*-value are presented on the top right of each graph.

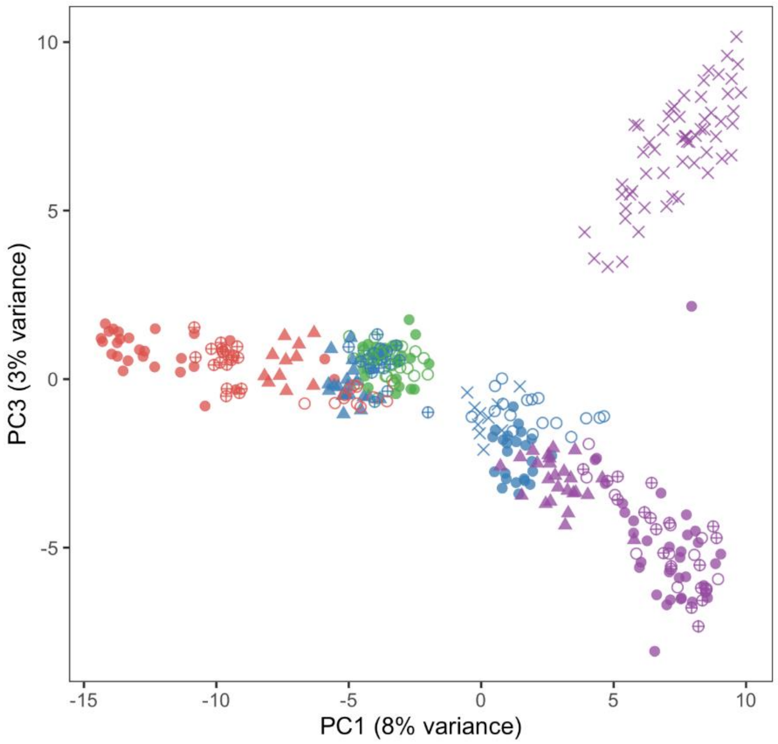

Figure S6 PCA analysis of the ALB populations in China. PC1 and PC3 explained 8% and 3% of the genetic variance, respectively. A total of 6,102 SNPs were involved in the analysis. For population colours and shapes, refer to Figure 2A.

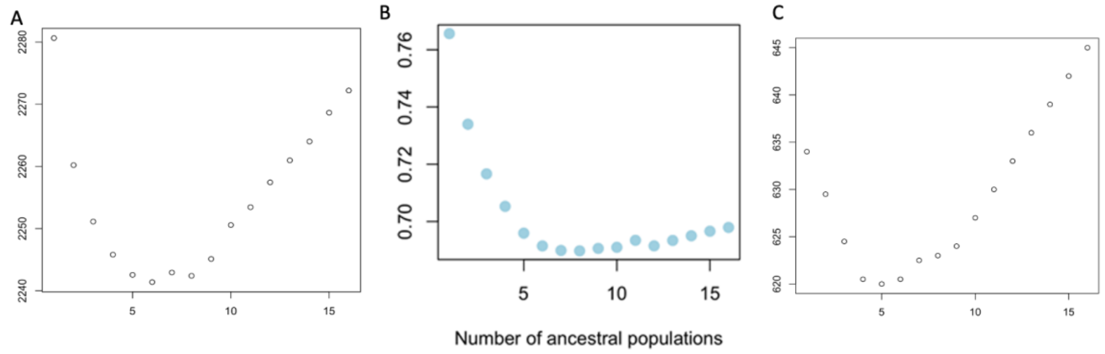

Figure S7 A Bayesian Information Criterion (BIC) for the SNP dataset used in the DAPC analysis. The x-axis indicates the number of clusters and the y-axis shows the BIC values. **B** The cross-entropy criterion (y-axis) for the respective number of ancestral populations (x-axis) as identified in the LEA analysis (SNP marker analysis only). **C** BIC for the microsatellite dataset used in the DAPC.

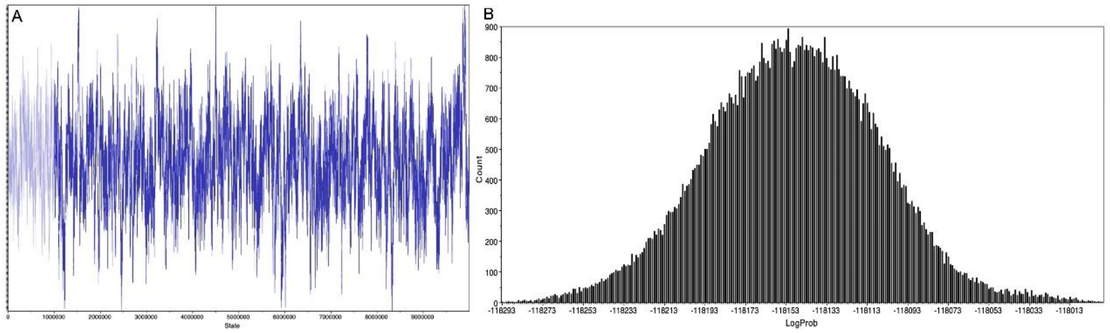

Figure S8 Trace file visualization of BayesAss results. **A** Trace plot of BayesAss analysis. **B** Posterior probability distribution.

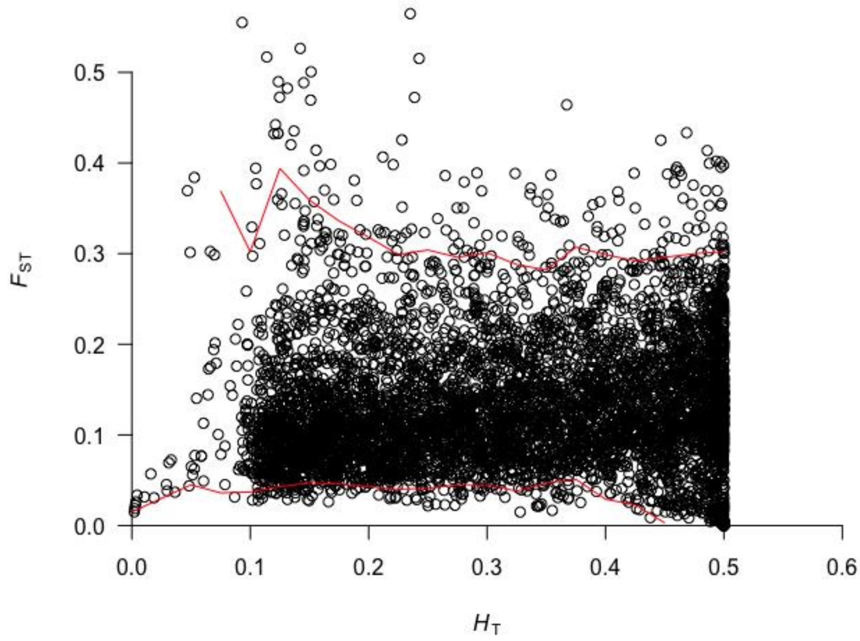

Figure S9 Heterozygosity (H_T)-F_{ST} relationship with smoothed quantiles in *fsthet*. Loci lying outside the quantiles were identified as outliers putatively under (positive or balancing) selection as determined under confidence intervals of 95% (red lines). The mean F_{ST} across all 6,102 loci is 0.134 ± 0.002.

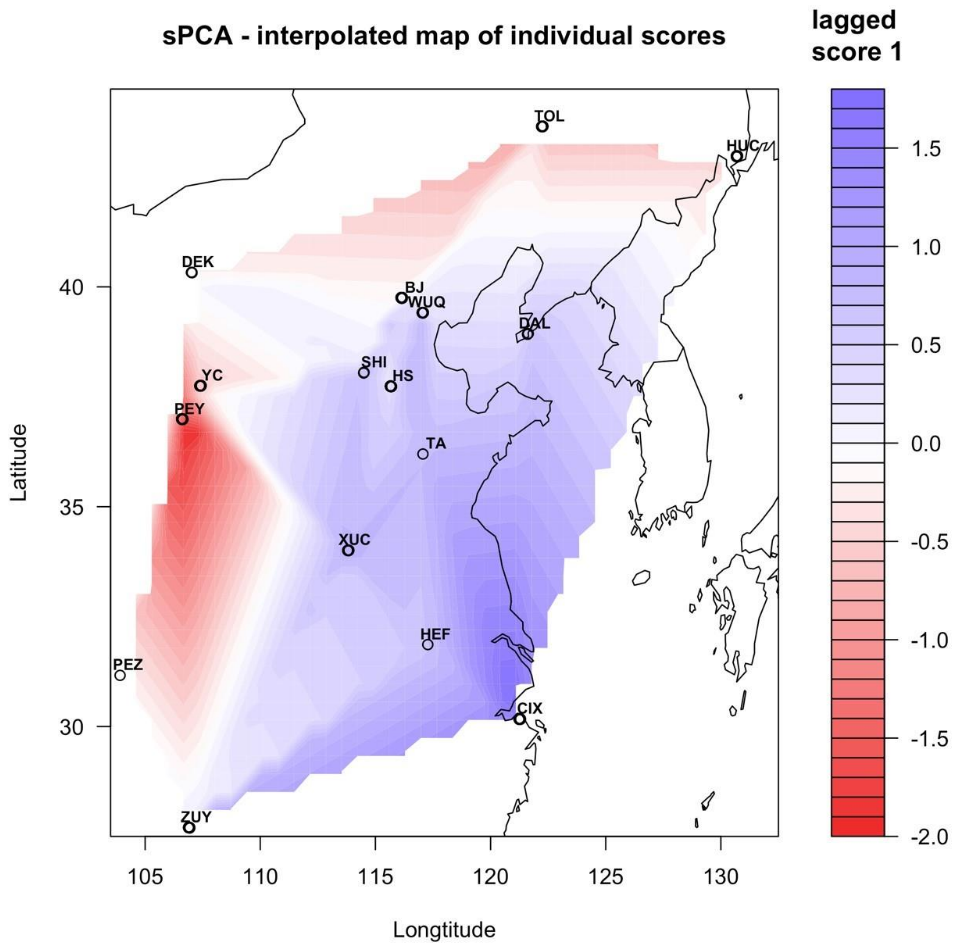

Figure S10 The sPCA analysis for the microsatellite dataset. The name codes for the populations are given in Table S3

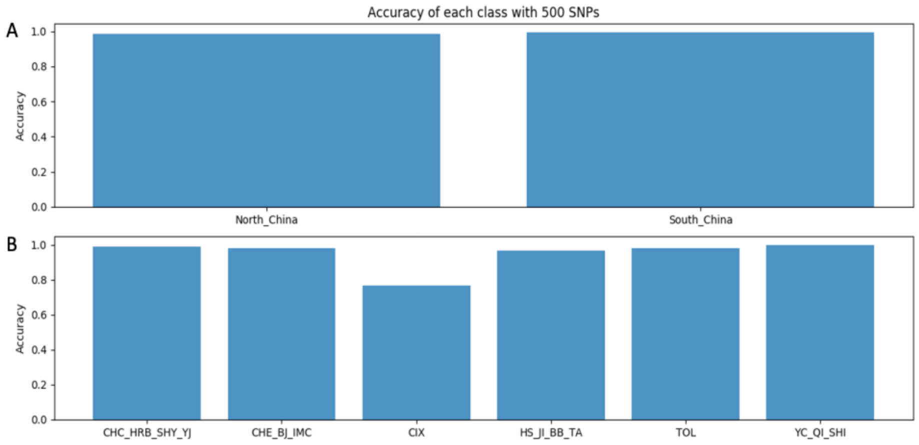

Figure S11 Assignment accuracy for each population assignment class in Mycorrhiza based on the most discriminant 500 SNPs (after testing the whole dataset of 6,102 SNPs). **A** ALB population divided into two groups (sPCA informed). **B** ALB populations divided into six groups (DAPC informed). Grouping information as in Figure S2.

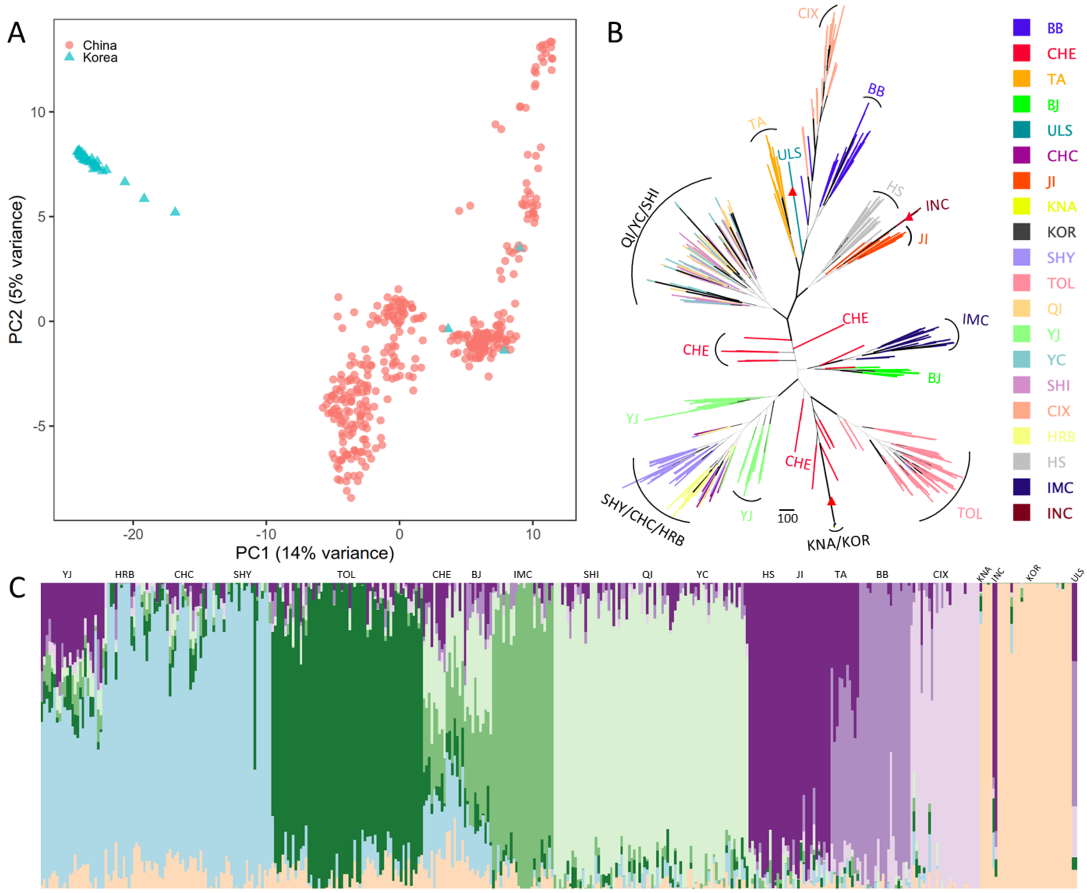

Figure S12 Population genetic analyses of native ALBs in China and Korea (including related Korean samples). **A** PCA analysis. **B** Maximum likelihood phylogenetic tree in RAxML. Each branch represents a sample. The width of each branch corresponds with the bootstrap value. The scale bar shows a bootstrap value of 100. Branches with a red triangle point to Korean samples. **C** Bar plot of ancestry coefficients identified in LEA (optimal K=8). Rotated labels indicate the Korean samples. A total of 6,102 SNPs were used in the analyses. For population codes, see Table 1. For relatedness estimates of KNA/KOR, see Table S12.
